# HIV-Tat Exacerbates the Actions of Atazanavir, Efavirenz, and Ritonavir on Cardiac Ryanodine Receptor (RyR2)

**DOI:** 10.3390/ijms24010274

**Published:** 2022-12-23

**Authors:** Fadhel A. Alomar, Chengju Tian, Sean R. Bidasee, Zachary L. Venn, Evan Schroder, Nicholas Y. Palermo, Mohammad AlShabeeb, Benson J. Edagwa, Jason J. Payne, Keshore R. Bidasee

**Affiliations:** 1 Department of Pharmacology and Toxicology, College of Clinical Pharmacy, Imam Abdulrahman Bin Faisal University, Dammam 31441, Saudi Arabia; 2 Departments of Pharmacology and Experimental Neuroscience, University of Nebraska Medical Center, Omaha, NE 68198, USA; 3 Vice Chancellor for Research Cores, University of Nebraska Medical Center, Omaha, NE 68198, USA; 4 Population Health Research Section, King Abdullah International Medical Research Center, King Saudi bin Abdulaziz University for Health Sciences, Riyadh 11426, Saudi Arabia; 5 Department of Internal Medicine, Division of Cardiovascular Medicine, University of Nebraska Medical Center, Omaha, NE 68198, USA; 6 Department of Environment and Occupational Health, University of Nebraska Medical Center, Omaha, NE 68198, USA; 7 Nebraska Redox Biology Center, Lincoln, NE 68588, USA

**Keywords:** sudden cardiac death, HIV, HIV-Tat, antiretroviral drugs, type 2 ryanodine receptor (RyR2), [^3^H]ryanodine, single-channel analysis

## Abstract

The incidence of sudden cardiac death (SCD) in people living with HIV infection (PLWH), especially those with inadequate viral suppression, is high and the reasons for this remain incompletely characterized. The timely opening and closing of type 2 ryanodine receptor (RyR2) is critical for ensuring rhythmic cardiac contraction–relaxation cycles, and the disruption of these processes can elicit Ca^2+^ waves, ventricular arrhythmias, and SCD. Herein, we show that the HIV protein Tat (HIV-Tat: 0–52 ng/mL) and therapeutic levels of the antiretroviral drugs atazanavir (ATV: 0–25,344 ng/mL), efavirenz (EFV: 0–11,376 ng/mL), and ritonavir (RTV: 0–25,956 ng/mL) bind to and modulate the opening and closing of RyR2. Abacavir (0–14,315 ng/mL), bictegravir (0–22,469 ng/mL), Rilpivirine (0–14,360 ng/mL), and tenofovir disoproxil fumarate (0–18,321 ng/mL) did not alter [^3^H]ryanodine binding to RyR2. Pretreating RyR2 with low HIV-Tat (14 ng/mL) potentiated the abilities of ATV and RTV to bind to open RyR2 and enhanced their ability to bind to EFV to close RyR2. In silico molecular docking using a Schrodinger Prime protein–protein docking algorithm identified three thermodynamically favored interacting sites for HIV-Tat on RyR2. The most favored site resides between amino acids (AA) 1702–1963; the second favored site resides between AA 467–1465, and the third site resides between AA 201–1816. Collectively, these new data show that HIV-Tat, ATV, EFV, and RTV can bind to and modulate the activity of RyR2 and that HIV-Tat can exacerbate the actions of ATV, EFV, and RTV on RyR2. Whether the modulation of RyR2 by these agents increases the risk of arrhythmias and SCD remains to be explored.

## 1. Introduction

The incidence of sudden cardiac death (SCD) in people living with HIV infection (PLWH), especially those with inadequate viral suppression, is significantly higher than that of the general population [1,2,3,4,5,6]. The risk factors that contribute to this include sex, vascular aging, hyperactive sympathetic and immune systems, inflammation, cardiac ischemia and fibrosis, and illicit and prescription drug use [1,5,6,7,8,9,10,11]. Data also suggest nonarrhythmic and arrhythmic causes for SCD in PLWH, with the former attributed to occult drug overdose [10]. To date, the arrhythmic causes of SCD have been linked to components of antiretroviral treatment with ischemic heart disease, and the inhibitory actions of HIV auxiliary proteins, select antiretrovirals, and other prescription medications, as well as recreational and illicit drug use causing the K^+^ channels to repolarize [1,5,12,13,14,15,16,17,18,19,20,21,22,23,24]. Delayed myocyte repolarization results in the prolongation of the QT interval, and a sudden tachycardia can lead to Torsade de Pointes, ventricular fibrillation, and SCD. To this end, clinicians have advocated for baseline and 12-lead ECG monitoring to be part of routine primary care for PLWH [25,26], along with antiretroviral medications that do not produce the clinically significant prolongation of the QT interval. These include the nucleotide reverse transcriptase inhibitors (tenofovir, abacavir, lamivudine, and emtricitabine), nonnucleoside reverse transcriptase inhibitors (efavirenz), and protease inhibitors (ritonavir and atazanavir) [1,4]. However, in a recent report based on post-SCD autopsy analyses, Tseng et al. [23] inferred that the association between QT-prolonging medications and death from arrhythmic causes may be overestimated in PLWH [16], suggesting that other mechanisms might be contributing to the high incidence of SCD in PLWH.

One likely cause is myocardial fibrosis [10,27]. Myocardial fibrosis, which arises from vascular leakage, ischemia, and tissue damage, can slow and/or block conduction through the myocardium, triggering re-entrant arrhythmias [27]. At high heart rates, cardiac fibrosis can lead to early after depolarizations (EADs), which are harbingers for atrial fibrillation (AF) [28] and ventricular fibrillation (VF) [29]. We also reported correlations between myocardial fibrosis, endothelial dysfunction microvasculature leakage, and ischemia in the hearts of HIV-1-infected humanized mice [30].

Another potential cause for ventricular fibrillation/arrhythmia and SCD is beat-to-beat alternations in the amplitude of evoked Ca^2+^ transients in myocytes, i.e., Ca^2+^ alternans [31,32]. One of the causes of Ca^2+^ alternans is a disruption to the timely opening and closing of type 2 ryanodine receptor (RyR2), which is the principal Ca^2+^-release channel on the SR involved in myocyte contraction [33,34,35]. RyR2s are activated (open) by a small amount of Ca^2+^ that enter myocytes following the depolarization-induced opening of L-type calcium channels to provide the Ca^2+^ needed for contraction. The elevated cytoplasmic Ca^2+^ and a sarcoplasmic reticulum (SR), depleted of Ca^2+^, serve as triggers to inactivate/close RyR2. The timely closing of RyR2 is critical during the reloading of the SR with Ca^2+^ in preparation for the next contraction cycle [36,37,38]. Prior studies have shown that mutations on RyR2 that lead to loss- or gain-of-function disrupt rhythmic SR Ca^2+^ cycling in myocytes. When heart rates increase in response to physical activity, emotional and other stressors, humans and animals with these mutations can also develop arrhythmias and even SCD (catecholaminergic polymorphic ventricular tachycardia) [39,40,41,42,43,44,45]. The activation or opening of RyR2 has also been identified as the underlying cause for the pro-rhythmics side effect of the antiarrhythmic drug flecainide [46,47,48].

To date, a limited number of studies have reported that HIV auxiliary proteins and select antiretroviral agents can bind to and modulate brain and skeletal muscle ryanodine receptors [47,49,50,51]. However, to the best of our knowledge, whether HIV proteins and antiretroviral drugs also modulate RyR2 remains poorly understood. Abacavir (ABC), atazanavir (ATV), bictegravir (BIC, an integrase strand transfer inhibitor), efavirenz (EFV), and tenofovir disoproxil fumarate (TDF) are widely used to treat HIV infection, especially in developing and emerging economies due to their high efficacy, tolerability, and low cost [52]. Rilpivirine (RPV) is another non-nucleoside reverse transcriptase inhibitor that is currently in use. Ritonavir is also compounded with ATV and lopinavir to increase the efficacy of protease inhibitor-based therapy [52]. These drugs are also not associated with clinically significant increases in the QT interval [1,4]. Thus, the objectives of this study were five-fold. First, to use a [^3^H]ryanodine binding assay to determine if the HIV transactivator protein Tat (HIV-Tat), ABC, ATV, BIC, EFV, tenofovir TDF, RPV, and RTV can modulate [^3^H]ryanodine binding to RyR2. Second, to use primary rat cardiac myocytes and live-cell confocal imaging to assess if agents that altered [^3^H]ryanodine binding to RyR2 can elicit ryanodine-sensitive Ca^2+^ waves. Third, to use a lipid bilayer single-channel assay to determine if agents that alter [^3^H]ryanodine binding to RyR2 and perturb Ca^2+^-homeostasis in myocytes exert their actions by directly modulating RyR2. Fourth, to determine if HIV-Tat can potentiate/attenuate the actions of the RyR2-modulating antiretroviral drugs. Fifth, to conduct in silico molecular docking using a Schrodinger Prime protein–protein docking algorithm to identify the potential sites of the interactions between HIV-Tat and RyR2.

## 2. Results

### 2.1. Effects of ABC, ATV, BIC, EFV, TDF, RPV, and RTV on Equilibrium [^3^H]ryanodine Binding to RyR2

An equilibrium [^3^H]ryanodine displacement assay offers a rapid and highly sensitive tool for screening compounds for their ability to interact with and modulate the activity of RyR2 [53,54]. The high-affinity binding site of ryanodine resides within the pore-forming region of RyR2, and the degree of “openness” of RyR2 can be adjusted by the amount of free [Ca^2+^] in the binding buffer. If a ligand binds to an open RyR2, then the amount of [^3^H]ryanodine bound at equilibrium will be enhanced. Conversely, if a ligand binds to and closes RyR2, then the amount of equilibrium [^3^H]ryanodine bound to RyR2 will be reduced. Ligands that do not alter equilibrium [^3^H]ryanodine binding to RyR2 are considered RyR2-inactive.

**Figure 1:**
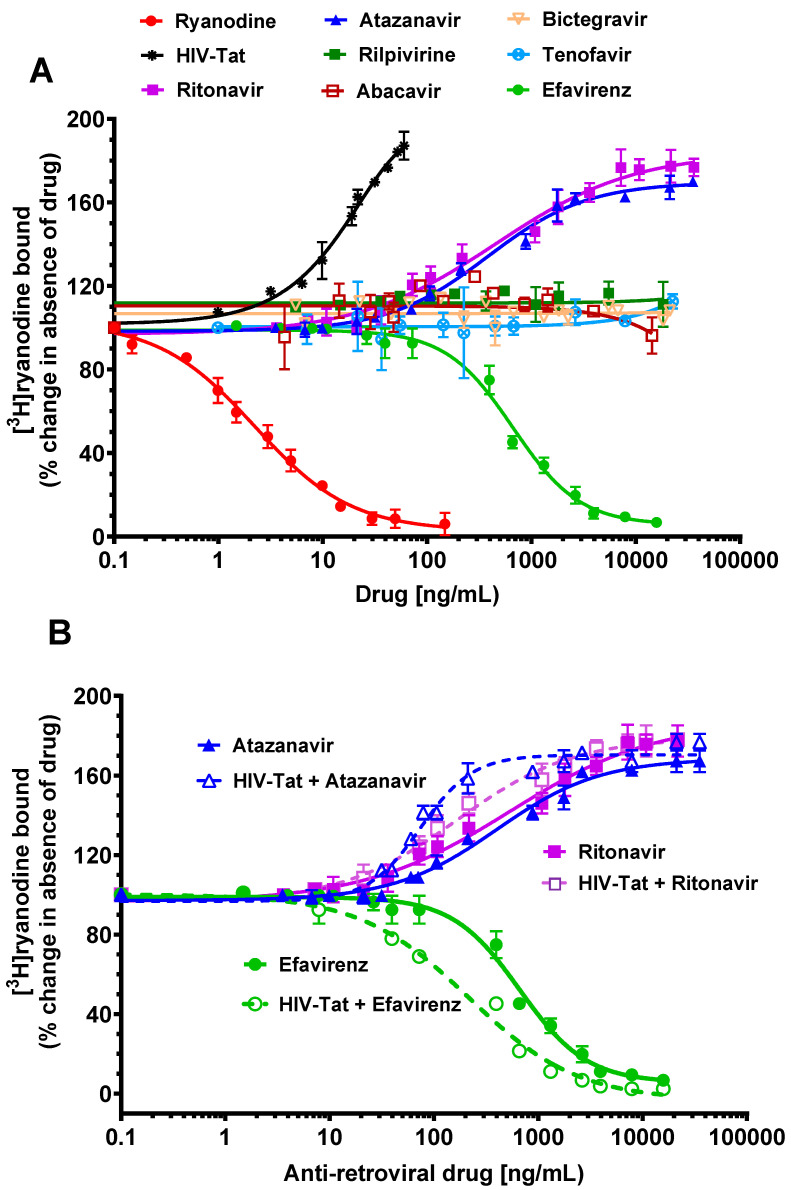
(**A**): Effects of Tat (HIV-Tat), abacavir (ABC)*,* atazanavir (ATV), bictegravir (BIC), efavirenz (EFV), tenofovir disoproxil fumarate (TDF), Rilpivirine (RPV), ritonavir (RTV), and the prototype ligand ryanodine on equilibrium [^3^H]ryanodine binding to RyR2. For these experiments, sarcoplasmic reticulum membrane vesicles isolated from bovine hearts were incubated in a buffer containing 500 mM KCl 20 mM Tris·HCl, 0.03 mM Ca^2+^, and 6.7 nM [^3^H]ryanodine, with a pH of 7.4 and with varying concentrations of HIV-Tat, ABC, ATV, BIC, EFV, TDF, RPV, RTV, and the prototype ligand ryanodine for 2 h at 37 °C. After this, the samples were filtered, and the [^3^H]ryanodine bound to filter paper was measured. The graphs show that HIV-Tat, ATV, and RTV dose-dependently enhanced equilibrium [^3^H]ryanodine binding to RyR2, while EFV dose-dependently attenuated equilibrium [^3^H]ryanodine binding to RyR2. ABC, BIC, and TDF, and RPV had no significant effect on equilibrium [^3^H]ryanodine binding to RyR2. The data shown for each compound are from n > 5 separate experiments carried out using at least three different sarcoplasmic reticulum membrane preparations. The curves were fitted using the binding analysis program Prism 9.0 (GraphPad Inc, San Diego, CA). (**B**): Effects of Tat (HIV-Tat) on equilibrium [^3^H]ryanodine binding to RyR2 by ATV, EFV, and RTV. For these experiments, sarcoplasmic reticulum membrane vesicles were incubated with HIV-Tat (7 ng/mL) for 10 min at 37 °C in a buffer containing 500 mM KCl 20 mM Tris·HCl, 0.03 mM Ca^2+^, and 6.7 nM [^3^H]ryanodine (pH 7.4). After this, varying concentrations of ATV, EFV, and RTV were added to the samples, and incubation continued for another 2 h at 37 °C. The samples were then filtered, and the [^3^H]ryanodine bound to filter paper was measured. The graphs show that the preincubation of sarcoplasmic reticulum vesicles with HIV-Tat shifted the equilibrium [^3^H]ryanodine binding curves of ATV, EFV, and RTV leftwards. Data shown are from n > 5 separate experiments done using at least three different sarcoplasmic reticulum membrane preparations. The curves were fitted using the binding analysis program Prism 9.0 (GraphPad Inc, San Diego, CA, USA).

The concentrations of ATV <35 ng/mL (<50 nM) had no quantitative effect on the amount of equilibrium [^3^H]ryanodine bound to RyR2. However, at higher concentrations (70–21,120 ng/mL, 0.1 to 30 μM), ATV potentiated the binding of equilibrium [^3^H]ryanodine to RyR2, reaching a maximum of 74.1 ± 5.2% over the control, with 21,120 ng/mL Figure 1A (▲). The data from the enhancement of [^3^H]ryanodine binding by ATV fitted to an [agonist] vs. response curve (variable slope with four parameters), with a r^2^ = 0.98. The EC_50_ was 400.4 ± 87.6 ng/mL (0.6 ± 0.1 μM), with a Hill slope of 0.9 ± 0.1.

The concentrations of EFV (<15 ng/mL (<50 nM)) also had no quantifiable effect on equilibrium [^3^H]ryanodine binding to RyR2. However, at concentrations between 31–18,912 ng/mL (0.1 to 60 µM), EFV dose-dependently decreased equilibrium [^3^H]ryanodine binding from RyR2 (Figure 1A (●). Maximal [^3^H]ryanodine displacement from RyR2 was achieved with 18,912 ng/mL EFV (60 µM). The displacement curve for EFV fitted to an [inhibitor] vs. response (variable slope; four parameters; r^2^ = 0.99). The IC_50_ was 663.4 ± 37.5 ng/mL (2.1 ± 0.01 μM), with a Hill slope of 1.3 ± 0.1. The displacement curve for the ryanodine fitted [agonist] vs. normalized response model was r^2^ = 0.99. Using the Cheng-Prussoff equation defined by K_i_ = IC_50_/(1 + (L/K_L_)) [55], where L is the concentration of the [^3^H]ryanodine used (6.7 nM), and K_L_ is the equilibrium dissociation constant of [^3^H]ryanodine = 1.2 nM for RyR2 [38,40], the *K*_i_ for EFV was 319.1 ± 15.7 nM. For a comparison, ryanodine (0.1–147 ng/mL) also displaced [^3^H]ryanodine from RyR2 at lower concentrations (Figure 1A (○).

The concentrations of RTV < 36 ng/mL (50 nM) also had no measurable effects on equilibrium [^3^H]ryanodine binding to RyR2. However, at higher concentrations (72–21,600 ng/mL, 0.1 to 30 μM), RTV potentiated equilibrium [^3^H]ryanodine binding to RyR2, reaching a maximum of 77.1 ± 5.2% over the control, with 21,600 ng/mL (Figure 1A) (■) The data from the [^3^H]ryanodine enhancement via RTV curve fitted to an [agonist] vs. response curve (four parameters), with an r^2^ = 0.96. The EC_50_ was 534.5.6 ± 254 ng/mL (0.75 ± 0.1 μM), with a Hill slope of 0.8 ± 0.1.

At concentrations of ≤1 ng/mL, HIV-Tat had no measurable effects on equilibrium [^3^H]ryanodine binding to RyR2. However, higher concentrations of HIV-Tat (1.5 ng/mL to 56 ng/mL) dose-dependently enhanced equilibrium [^3^H]ryanodine binding to RyR2, reaching a maximum percent enhancement of 85 ± 3.2%, with 56 ng/mL HIV-Tat, (Figure 1A (∗). The data fitted for THE HIV-Tat enhancement curve fitted to an [agonist] vs. response curve (four parameters) with r^2^ = 0.98. The EC_50_ was 22.7 ± 3.5 ng/mL, with a Hill slope was 1.1 ± 0.2. Heat-inactivated HIV-Tat had no effect on the binding of [^3^H]ryanodine to RyR2.

ABC (0–14,315 ng/mL), BIC (0–22,469 ng/mL), TDF (0–18,321 ng/mL), and RPV (0–14,360 ng/mL) had no significant effect on equilibrium [^3^H]ryanodine binding to RyR2 (Figure 1A).

### 2.2. Effects of Pretreating RyR2 with HIV-Tat on Equilibrium [^3^H]ryanodine Binding to ATV, RTV, and EFV

Having found that ATV, RTV, EFV, and HIV-Tat alter equilibrium [^3^H]ryanodine binding to RyR2, we then investigated if pretreating RyR2 with a low HIV-Tat to mimic inadequate HIV-suppression would alter the ability of ATV, EFV, and RTV to modulate [^3^H]ryanodine binding to RyR2.

Preincubating junctional SR vesicles with 14 ng/mL HIV-Tat for 10 min at 37 °C prior to the addition of ATV, EFV, or RTV resulted in leftward shifts in their equilibrium [^3^H]ryanodine binding curves. The EC_50_ for ATV in the presence of HIV-Tat was 5.3-fold lower than that in the absence of HIV-Tat (70.8 ± 17.6 ng/mL; 0.1 ± 0.0 μM; Figure 1B (Δ)), with an increase in the Hill slope (1.7 ± 0.1). The EC_50_ for RTV in the presence of HIV-Tat was 3.1-fold lower than that in the absence of HIV-Tat (167.3 ± 29.6 ng/mL; 0.08 ± 0.01 μM; Figure 1B (□)), with no change in the Hill slope (0.8 ± 0.1). The IC_50_ for EFV in the presence of HIV-Tat was also 3.1-fold lower than that in the absence of HIV-Tat (214.5 ± 37.5 ng/mL; 0.7 ± 0.1 μM; Figure 1B (○)), with a decrease in the Hill slope (0.9 ± 0.1). These data indicate that when HIV-Tat is present in the binding medium, the affinities of ATV, EFV, and RTV for RyR2 are enhanced.

### 2.3. Effects of ATV, EFV, RTV, and HIV-Tat on Ca^2+^ Homeostasis in Primary Rat Cardiac Myocytes

Since ATV, EFV, RTV, and HIV-Tat modulated the equilibrium binding of [^3^H]ryanodine to RyR2, we then investigated if ATV, EFV, RTV, and HIV-Tat could also elicit Ca^2+^ transients/waves in rat primary cardiac myocytes. For this, live-cell confocal imaging was conducted with freshly isolated rats that were loaded with the Ca^2+^-sensitive dye Fluo-3.

**Figure 2:**
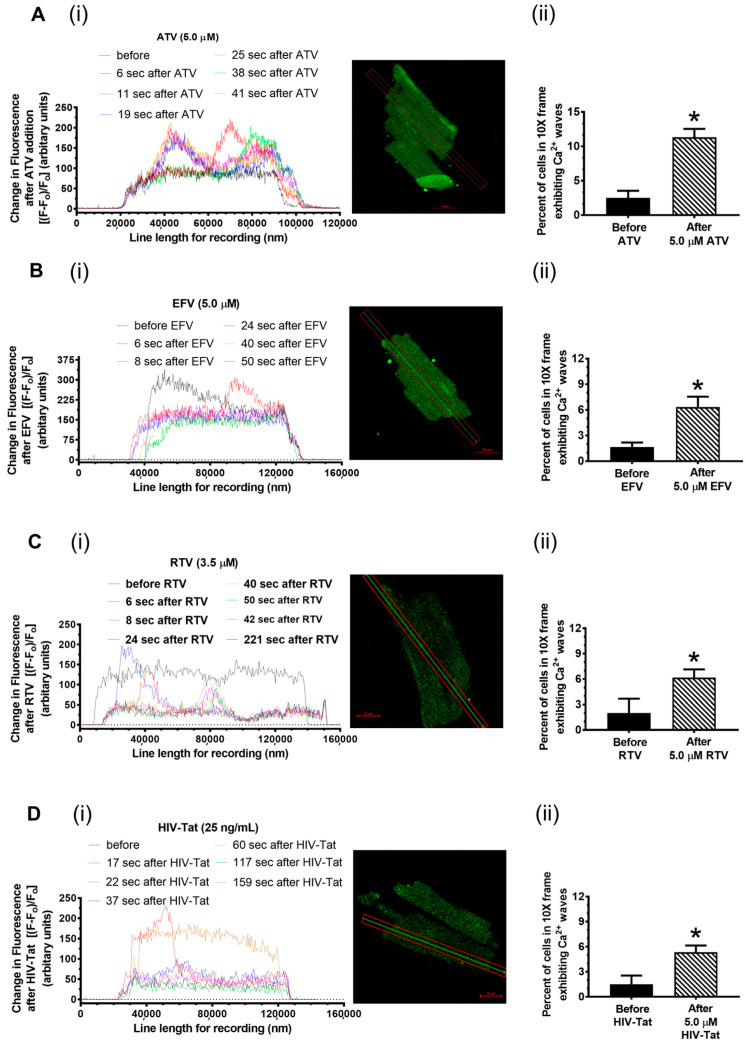
Time-dependent changes in intracellular Ca^2+^ in primary rat ventricular myocytes following the addition of a bolus dose of HIV-Tat, ATV, EFV, and RTV. For this, primary ventricular myocytes isolated from rat hearts were incubated with the Ca^2+^-sensitive Fluo-3; these were placed on the head stage of a laser confocal microscope equipped with 10× and 40× objectives (Zeiss Confocal LSM 710, equipped with an Argon-Krypton Laser, 25 mW argon laser, 2% intensity, Thornwood, NJ, excitation wavelength 488 nm, and emission wavelengths of 515 nm). **Panel A(i)** shows the time-dependent changes in intracellular Ca^2+^ from a representative rat ventricular myocyte when exposed to ATV (5.0 µM). **Panel A(ii)** shows the mean ± SD for >40 cells in the four separate chambers before and after ATV treatment. **Panel B(i)** shows the time-dependent changes in intracellular Ca^2+^ from a representative rat ventricular myocyte when exposed to EFV (5.0 µM). **Panel B(ii)** shows the mean ± SD for >40 cells in the four separate chambers before and after EFV treatment. **Panel C(i)** shows the time-dependent changes in intracellular Ca^2+^ from a representative rat ventricular myocyte when exposed to RTV (5.0 µM). **Panel C(ii)** shows the mean ± SD for >40 cells in the four separate chambers before and after RTV treatment. **Panel D(i)** shows the time-dependent changes in intracellular Ca^2+^ from a representative rat ventricular myocyte when exposed to HIV-Tat (25 ng/mL). **Panel D(ii)** shows the mean ± SD for >40 cells in four separate chambers before and after HIV-Tat treatment. * denotes significantly different (*p* < 0.05) from the control (before the addition of the drug).

When antiretroviral-naïve primary rat ventricular myocytes were exposed to 5.0 μM ATV, Ca^2+^ transients/waves were seen within one minute after the addition of the drug. The Ca^2+^ waves elicited by ATV persisted for the duration of the recording (60 s). The myocyte contractions and relaxations repeated during the recording. In the four chambers of cells (>10 myocytes per 10× frame per chamber) investigated, the Ca^2+^ waves and contraction/relaxation stopped 240 s after ATV exposure. Figure 2A(i) shows the ATV-induced-Ca^2+^ waves in a representative myocyte (also see Supplemental Video S1 (60 s of recording)). Figure 2A(ii) shows a summary of the cells in the four separate chambers before and after ATV treatment. Pretreating the antiretroviral-naïve myocytes with 50 µM for 10 min to block RyR2 significantly attenuated the Ca^2+^ waves induced by ATV (see Supplemental Video S2 (60 s of recording)), suggesting that ATV elicits Ca^2+^ release from the SR.

Exposing antiretroviral-naïve primary rat ventricular myocytes to 5.0 μM EFV also leads to Ca^2+^ transients/waves within one minute of being administered. In the four chambers of the cells (>10 myocytes per 10× frame per chamber) investigated, the Ca^2+^ waves and contraction/relaxation stopped 140–150 s after the addition of EFV. The myocytes exposed to EFV also showed elevated Ca^2+^ originating from regions close to the plasmalemma prior to “permanent cell shortening” within 60 s after EFV addition to the culture medium. Figure 2B(i) shows the EFV-induced Ca^2+^ waves in a representative myocyte (also see Supplemental Video S3 (60 s of recording)). It is of interest to note that while the number of Ca^2+^ waves elicited by EFV (per unit of time) reduced when compared to ATV, their amplitudes were 25 ± 5% more than that of ATV (comparing Figure 2A(ii) and B(ii)). Figure 2B(ii) shows a summary of the data of the cells in the four separate chambers before and after EFV treatment that exhibited the Ca^2+^ waves. Pretreating antiretroviral-naïve myocytes with 50 µM for 10 min to block RyR2 significantly attenuated the Ca^2+^ waves induced by EFV (see Supplemental Video S4 (60 s of recording)). However, we did notice an elevation in Ca^2+^ originating from those regions close to the plasmalemma of the myocytes prior to “permanent cell shortening”, suggesting that EFV elevates cytoplasmic Ca^2+^ by nonryanodine receptor mechanisms as well.

Exposing antiretroviral-naïve primary rat ventricular myocytes to 5.0 μM RTV also resulted in Ca^2+^ transients. However, the pattern of Ca^2+^ elevation was distinct from that of ATV and EFV. After the addition of RTV, the cytosolic Ca^2+^ in most of the myocytes in the four chambers investigated (>10 myocytes per 10× frame per chamber) increased. But the increase was in multiple distinct regions throughout the myocyte without significant Ca^2+^ wave generation, which is reminiscent of increases in spontaneous Ca^2+^ sparks. This was followed by global Ca^2+^ transients with and without wave formation. Figure 2C(i) shows the RTV-induced Ca^2+^ waves in a representative myocyte (also see Supplemental Video S5, (240 s of recording)). Figure 2C(ii) shows the summary data of the cells in the four separate chambers before and after RTV treatment that exhibited Ca^2+^ waves. Pretreating antiretroviral-naïve myocytes with 50 µM for 10 min to block RyR2 significantly attenuated the Ca^2+^ waves induced by RTV (see Supplemental Video S6 (120 s of recording)).

Exposing antiretroviral-naïve primary rat ventricular myocytes to 25 ng/mL HIV-Tat resulted in Ca^2+^ transients/waves within one minute after administration to the culture medium in the four chambers of the cells (>10 myocytes per 10× frame per chamber) investigated. In many of the cells, HIV-Tat also elicited a global elevation in myocyte Ca^2+^, which was followed by irreversible cell shortening. Figure 2D(i) shows the HIV-induced Ca^2+^ waves in a representative myocyte (also see Supplemental Video S7, (240 s of recording)). Figure 2D(ii) shows the summary data of the cells in the four separate chambers of the myocytes before and after HIV-Tat treatment. Pretreating antiretroviral-naïve myocytes with 50 µM for 10 min to block RyR2 significantly attenuated the Ca^2+^ waves induced by HIV-Tat (see Supplemental Video S8). Heat-inactivated HIV-Tat did not alter Ca^2+^ homeostasis in rat ventricular myocytes.

### 2.4. Effects of ATV. EFV, RTV, and HIV-Tat on the Gating and Conductance of RyR2

Next, single-channel studies were conducted to gain insights into the effects of ATV, EFV, RTV, and HIV-Tat on the gating and conductance of RyR2. In this study, all agents were added cumulatively to the *cis* chamber of the bilayer, equivalent to the cytosolic side of RyR2.

#### 2.4.1. Effects of ATV on RyR2

**Figure 3:**
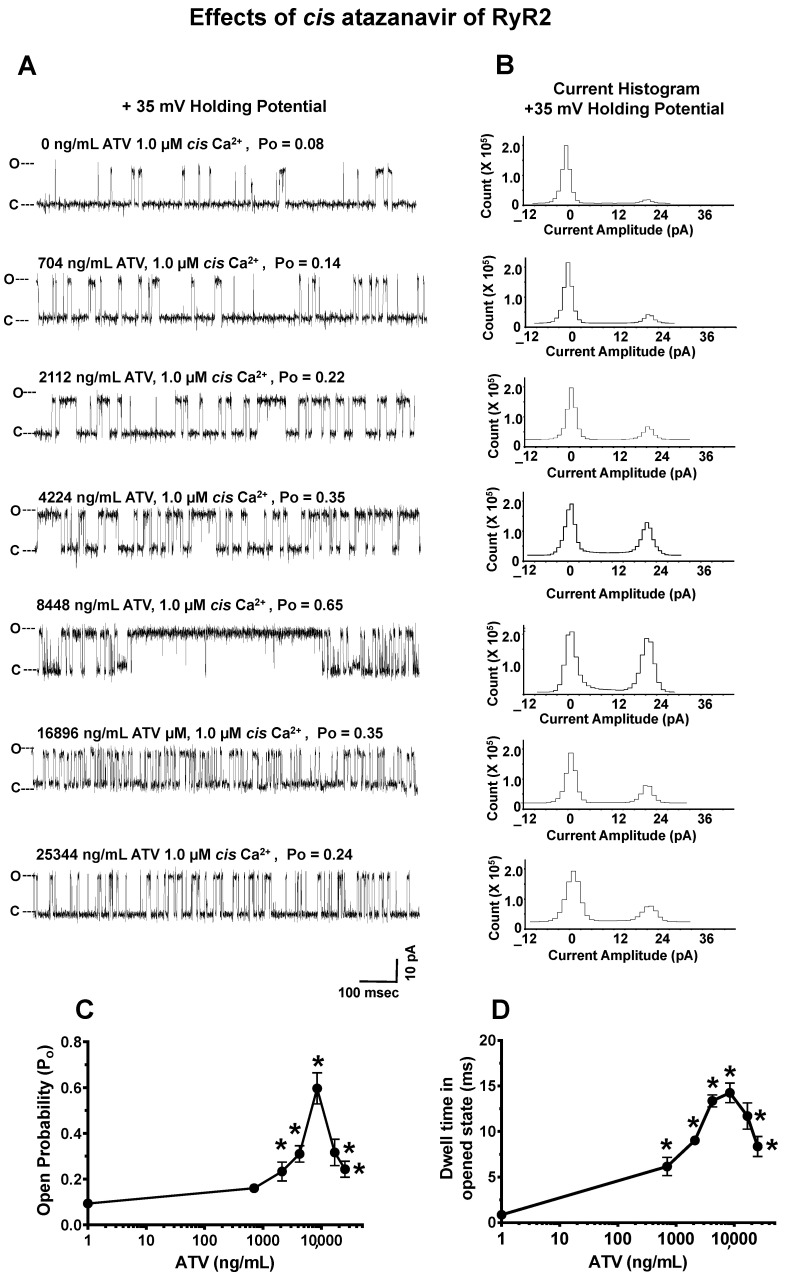
Effects of increasing cis concentrations of atazanavir (ATV) on the open probability of RyR2. **Panel A** shows a representative 1.0 s recording of a RyR2 channel with 1.0 µM *cis* Ca^2+^ and cumulative amounts of *cis* ATV. **Panel B** shows the current histograms for the channel with cumulative amounts of *cis* ATV. **Panels C**,**D** are graphs of the open probability and dwell time (mean ± SD) of RyR2 as a function of the *cis* concentrations of the ATV channels for n = 13 channels. The recordings shown are at +35 mV (upward deflections) in a symmetric KCl buffer solution (0.25 mM KCl, 20 mM K/HEPES, pH 7.4). O, open; C, closed. * denotes significantly different (*p* < 0.05) from the control (before the addition of the drug).

The mean open probability (P_o_) and current amplitude of the RyR2 channels used prior to the addition of ATV was 0.08 ± 0.01 and 21.4 ± 1.3 pA (a representative channel is shown in Figure 3; conductance = 712 ± 36 pS; 1.0 μM *cis* Ca^2+^ at +35 mV holding potential (HP); n = 13 channels, respectively). These channels were kept at a low P_o_ to assess the ability of ATV to both open and close RyR2. The cumulative addition of ATV to the *cis* chamber (704–8448 ng/mL, 1.0–12.0 µM) dose-dependently increased the P_o_ of RyR2 from 0.08 ± 0.03 to 0.65 ± 0.11 within 15 s of addition (Figure 3A–C). The increase in P_o_ arose principally from an increase in the dwell time in the open state, with no significant change in the current amplitude (Figure 3D). The concentrations of ATV (<704 ng/mL) and the solvent (1% ethanol) had no measurable effects on the P_o_ of RyR2 up to 15 m after their addition to the *cis* chamber. At 8448 ng/mL ATV, the dwell time in the open state for 10 out of the 13 channels lasted >500 ms (Figure 3A; fifth recording from top). Higher ATV concentrations (16,896–25,344 ng/mL) progressively decreased the P_o_ of RyR2 from 0.65 ± 0.11 to 0.24 ± 0.08 by decreasing the dwell time in the open state of the channels (Figure 3A–D).

#### 2.4.2. Effects of EFV on RyR2

**Figure 4:**
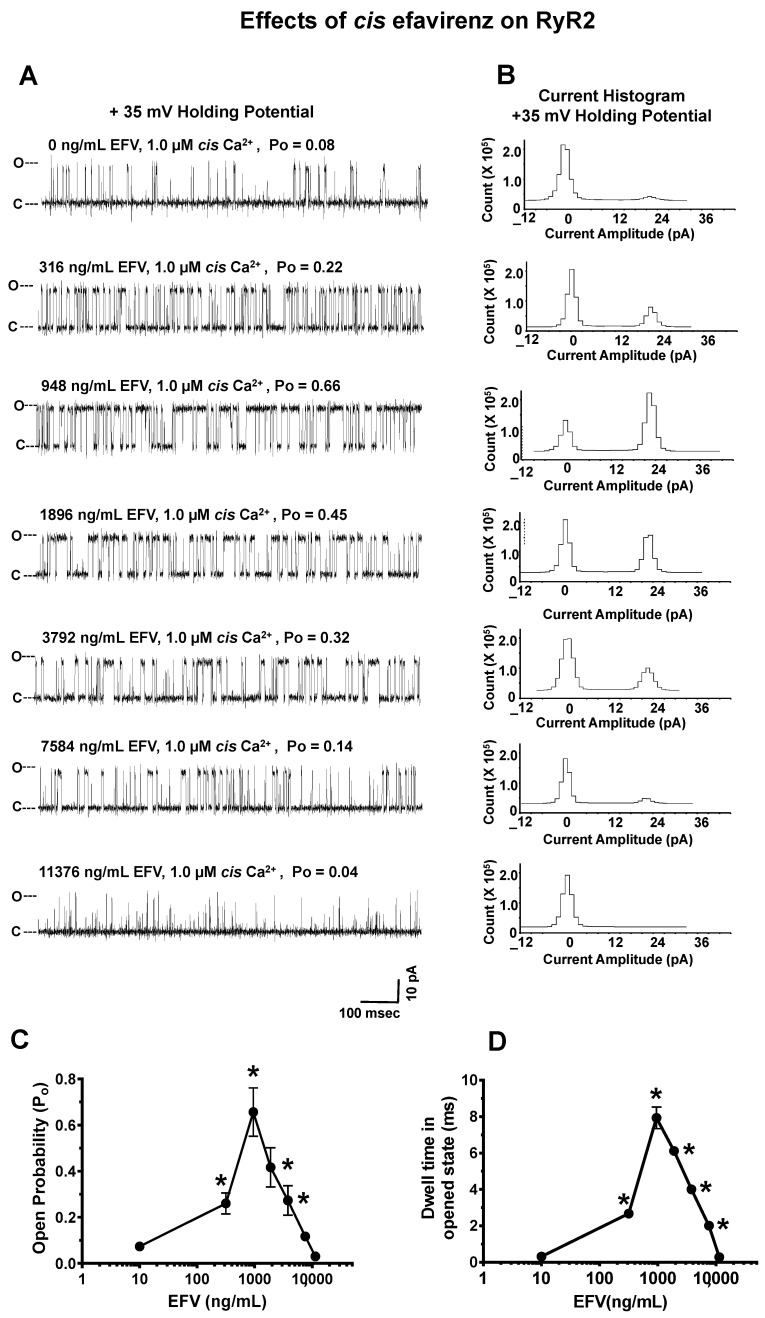
Effects of increasing *cis* concentrations of efavirenz (EFV) on the open probability of RyR2. **Panel A** shows a representative 1.0 s recording of a RyR2 channel with 1.0 µM *cis* Ca^2+^ and cumulative amounts of *cis* EFV. **Panel B** shows the current histograms for the channel with cumulative amounts of *cis* EFV. **Panels C**,**D** are graphs of the open probability and dwell time (mean ± SD) of RyR2 as a function of the *cis* concentrations of the EFV channels for n = 12 channels. Recordings shown are at +35 mV (upward deflections) in a symmetric KCl buffer solution (0.25 mM KCl; 20 mM K/HEPES; pH 7.4). O, open; C, closed. * denotes significantly different (*p* < 0.05) from the control (before the addition of the drug).

The mean P_o_ of RyR2 with 1.0 μM *cis* Ca^2+^ prior to the addition of *cis* EFV was 0.08 ± 0.02 at +35 mV HP (a representative channel is shown in Figure 3A; top). The mean current amplitude was 21.4 ± 1.6 pA (Figure 4B), which is equivalent to a conductance = 712 ± 56 pS. The addition of 316 ng/mL (1.0 µM) EFV to the *cis* chamber increased the P_o_ of RyR2 (within 15 s of addition) to 0.22, a 2.5-fold over basal (Figure 4A; second panel from top; Figure 4B,C). This increase in the P_o_ of RyR2 arose from an increase in the number of transitions from the close and fully open states (i.e., gating frequency) and from an increase in the dwell time in the open state (Figure 4C,D). The concentrations of EFV < 316 ng/mL and the solvent (1% ethanol) had no effect on the P_o_ of RyR2 up to 10 min after their addition. Increasing *cis* EFV to 948 ng/mL increased the P_o_ of RyR2 further to 0.66, primarily by increasing the dwell time in the open state (Figure 4A–D). Higher concentrations of EFV dose-dependently decreased the P_o_ of RyR2 by reducing the dwell time in the open state.

#### 2.4.3. Effects of RTV on RyR2

**Figure 5:**
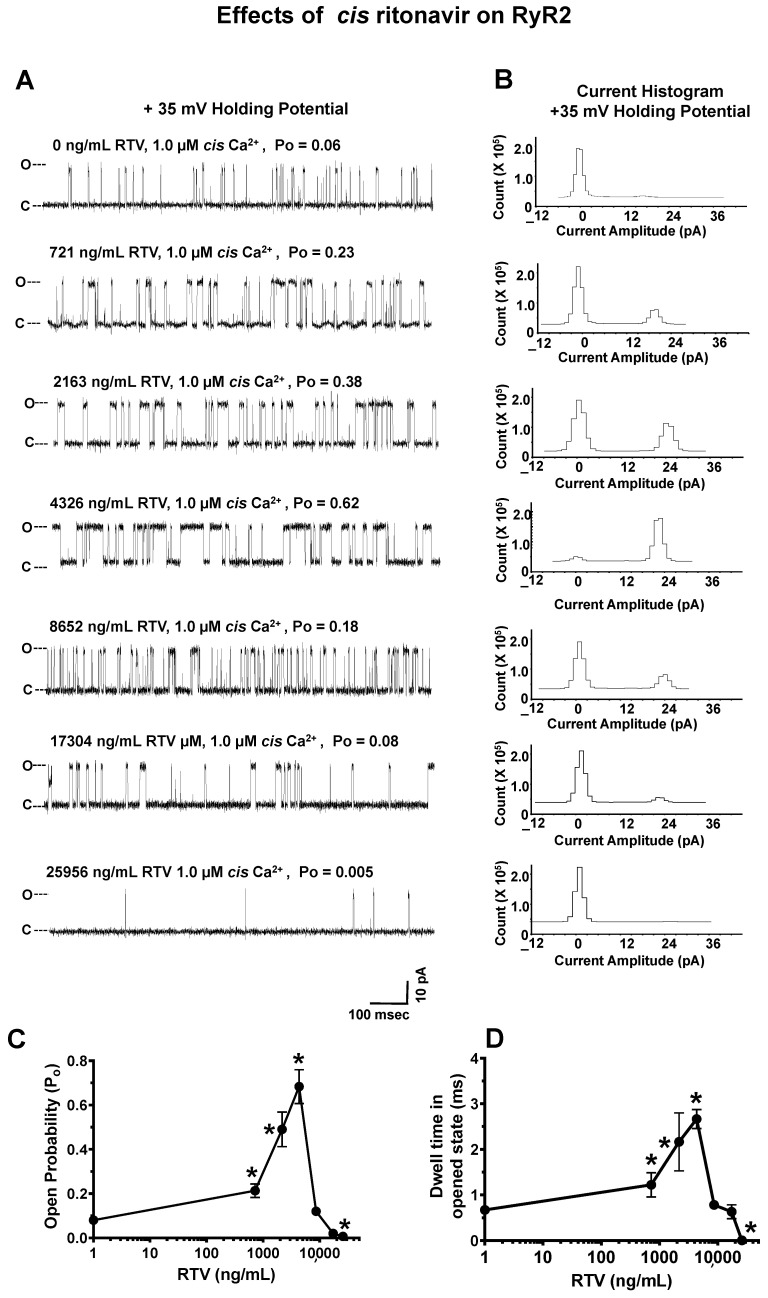
Effects of increasing *cis* concentrations of ritonavir (RTV) on the open probability of RyR2. **Panel A** shows a representative 1.0 s recording of a RyR2 channel with 1.0 µM *cis* Ca^2+^ and cumulative amounts of *cis* RTV; **Panel B** shows the current histograms for the channel with cumulative amounts of *cis* EFV. **Panels C**,**D** are graphs of the open probability and dwell time (mean ± SD) of RyR2 as a function of the *cis* concentrations of the RTV channels for n = 11 channels. Recordings shown are at +35 mV (upward deflections) in a symmetric KCl buffer solution (0.25 mM KCl; 20 mM K/HEPES; pH 7.4). O, open; C, closed. * denotes significantly different (*p* < 0.05) from the control (before the addition of the drug).

The mean P_o_ of the 11 RyR2 channels before the *cis* addition of RTV was 0.06 ± 0.01 at +35 mV HP, and their mean current amplitude was 21.5 ± 1.4 pA, which is equivalent to a conductance = 715.5 ± 45 pS (a representative channel is shown in Figure 4A). The addition of 721 ng/mL (1.0 µM) RTV to the 1.0 μM *cis* Ca^2+^ chamber increased the P_o_ of RyR2 3.8-fold to 0.20. The increase in P_o_ arose from an increase in the dwell time in the opened state (Figure 4D). Lower concentrations of RTV and the solvent (1% ethanol) did not have any quantitative effect on the P_o_ of RyR2. Increasing the *cis* concentration of RTV to 2163 and 4326 ng/mL increased the P_o_ of RyR2 further to 0.46 ± 0.05 and 0.82 ± 0.06, respectively, by increasing the dwell time in the opened state (Figure 5A–D). Higher concentrations of RTV in the *cis* chamber (8652–25,956 ng/mL) dose-dependently decreased the P_o_ of RyR2 to closure (0.006 ± 0.001; Figure 5A–D).

#### 2.4.4. Effects of HIV-Tat on RyR2

**Figure 6:**
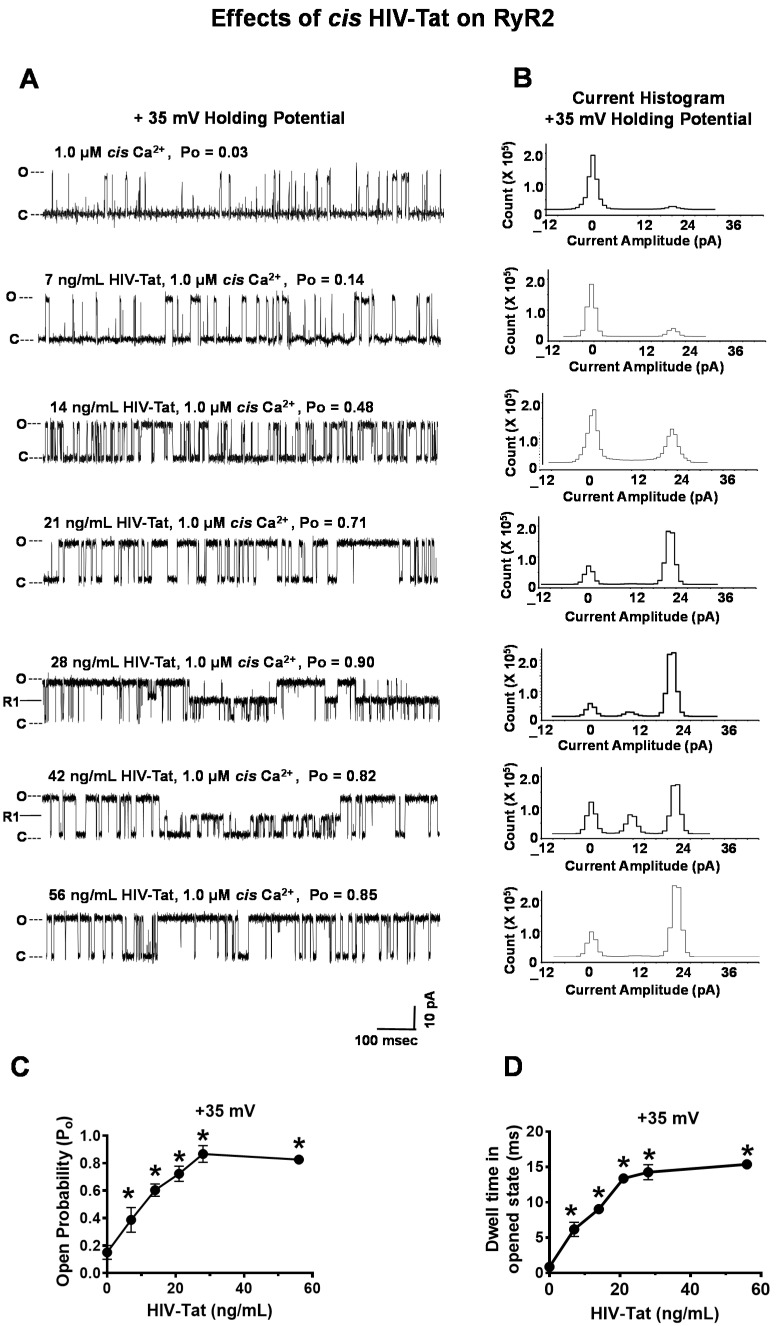
Effects of increasing *cis* concentrations of HIV-Tat on the open probability of RyR2. **Panel A** shows a representative 1.0 s recording of a RyR2 channel with 1.0 µM *cis* Ca^2+^ and cumulative amounts of *cis* HIV-Tat. **Panel B** shows the current histograms for the channel with cumulative amounts of *cis* EFV. **Panels C**,**D** are graphs of the open probability and dwell time of RyR2 (mean ± SD) as a function of the *cis* concentrations of the HIV-Tat channels for n = 10 channels. Recordings shown are at +35 mV (upward deflections) in a symmetric KCl buffer solution (0.25 mM KCl; 20 mM K/HEPES; pH 7.4). O, open; C, closed. * denotes significantly different (*p* < 0.05) from the control (before the addition of the drug).

The mean P_o_ of the RyR2 channels with 1.0 μM *cis* Ca^2+^ at + 35 mV (HP) was 0.03 ± 0.00 (a representative channel is shown in Figure 6A; top). The mean current amplitude was 21.8 ± 1.8 pA, which is equivalent to a conductance of 726 ± 60 pS. The addition of HIV-Tat (7 ng/mL) to the *cis* chamber increased the P_o_ of RyR2 to 0.14 ± 0.01 at + 35 mV HP in 10 out of the 11 channels, respectively (Figure 6A–C). The other channel did not respond to Ca^2+^ or HIV-Tat. The increase in P_o_ via 7 ng/mL of *cis* HIV-Tat arose from an increase in the frequency of the transitions from the closed to fully open states (gating frequency) and the dwell time in the open state (Figure 6A,D). The solvent used (1% ethanol) and HIV-Tat <7ng/mL had no measurable change on the P_o_ of RyR2 up to 10 min. Cumulatively increasing *cis* HIV-Tat to 56 ng/mL dose-dependently increased the P_o_ of RyR2 to + 35 mV HP (Figure 6A–D). At these higher concentrations, the increase in P_o_ arose primarily from an increase in the dwell time in the open state (Figure 6D). At 28 and 42 ng/mL HIV-Tat, a reversible state was induced from the reduced conductance (R1) (50% of maximum; 346 ± 15 pS) that lasted for ≥ 200 ms (Figure 6A). At 56 ng/mL *cis*, HIV-Tat increased the P_o_ of RyR2 up to 0.85 ± 0.05 at + 35mV HP. Heat-inactivated HIV-Tat (boiled for 10 m) at 7–42 ng/mL had no quantitative effects on the P_o_ of RyR2 (Supplemental Figure S1). However, heat-inactivated HIV-Tat at 56 ng/mL decreased the conductance of RyR2 (Supplemental Figure S1). We suspect that this may be due, in part, to heat-inactivated HIV-Tat nonspecifically interacting with the pores of RyR2.

#### 2.4.5. Effects of Luminal Ca^2+^ on HIV-Tat Actions on RyR2

**Figure 7:**
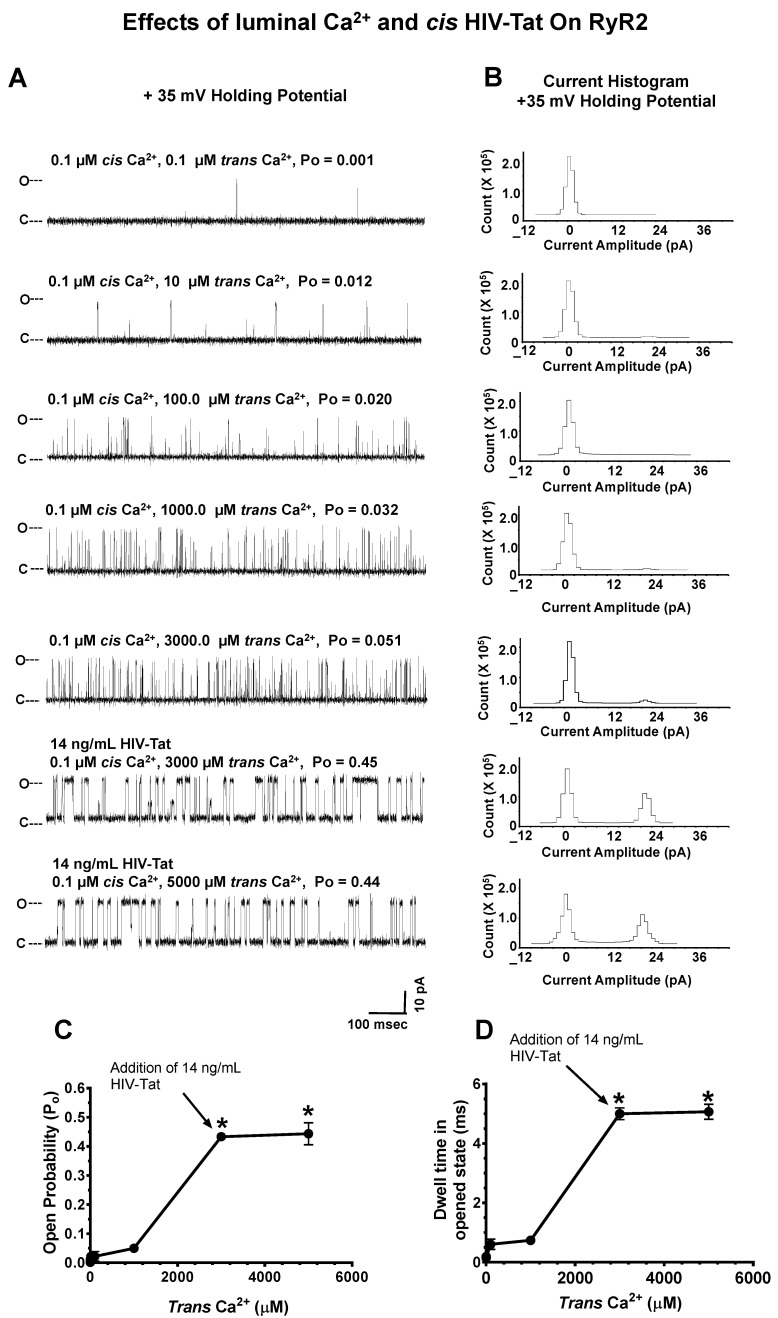
Effects of luminal Ca^2+^ and *cis* HIV-Tat on the open probability of RyR2. **Panel A** shows a representative 1.0 s recording of a RyR2 channel with 0.1 µM *cis* Ca^2+^ and increasing amounts of *trans* Ca^2+^. The lower two recordings occurred with 14 ng/mL *cis* HIV-Tat with 3000 μM and 5000 μM trans Ca^2+^. **Panel B** shows the current histograms for the channel with cumulative amounts of *cis* EFV. **Panels C**,**D** are graphs of the open probability and dwell time (mean ± SD) of RyR2 as a function of the *cis* concentrations of the HIV-Tat channels for n = 12 channels. Recordings shown are at +35 mV (upward deflections) in a symmetric KCl buffer solution (0.25 mM KCl; 20 mM K/HEPES; pH 7.4). O, open; C, closed. * denotes significantly different (*p* < 0.05) from the control (before the addition of the drug).

We also investigated whether the ability of HIV-Tat to activate RyR2 was dependent on *trans* Ca^2+^ content (i.e., the SR Ca^2+^ content). In this experiment, when *cis* Ca^2+^ was kept at 100 nM, and when and *trans* Ca^2+^ was progressively increased from 100 nM to 3000 µM, the P_o_ of RyR2 increased from 0.001 to 0.051 (a representative channel is shown in Figure 7). The increase in P_o_ was significant (*p* < 0.05) and arose from increases in the gating frequency and not dwell times in the opened state (0.22 ± 0.01 ms). The addition of 14 ng/mL HIV-Tat to the *cis* chamber increased the P_o_ to 0.45, similar to that with a low *trans* Ca^2+^ (Figure 7A: third panel from the top; see also Figure 5A: third panel from top). Increasing *trans* Ca^2+^ further to 5000 µM in the presence of 14 ng/mL *cis* HIV-Tat did not increase the P_o_ of RyR2 further. These data suggest that the ability of HIV-Tat to activate RyR2 was independent of the SR Ca^2+^ content.

### 2.5. Effects of HIV-Tat Pretreatment on the Actions of ATV, EFV, and RTV on RyR2

**Figure 8:**
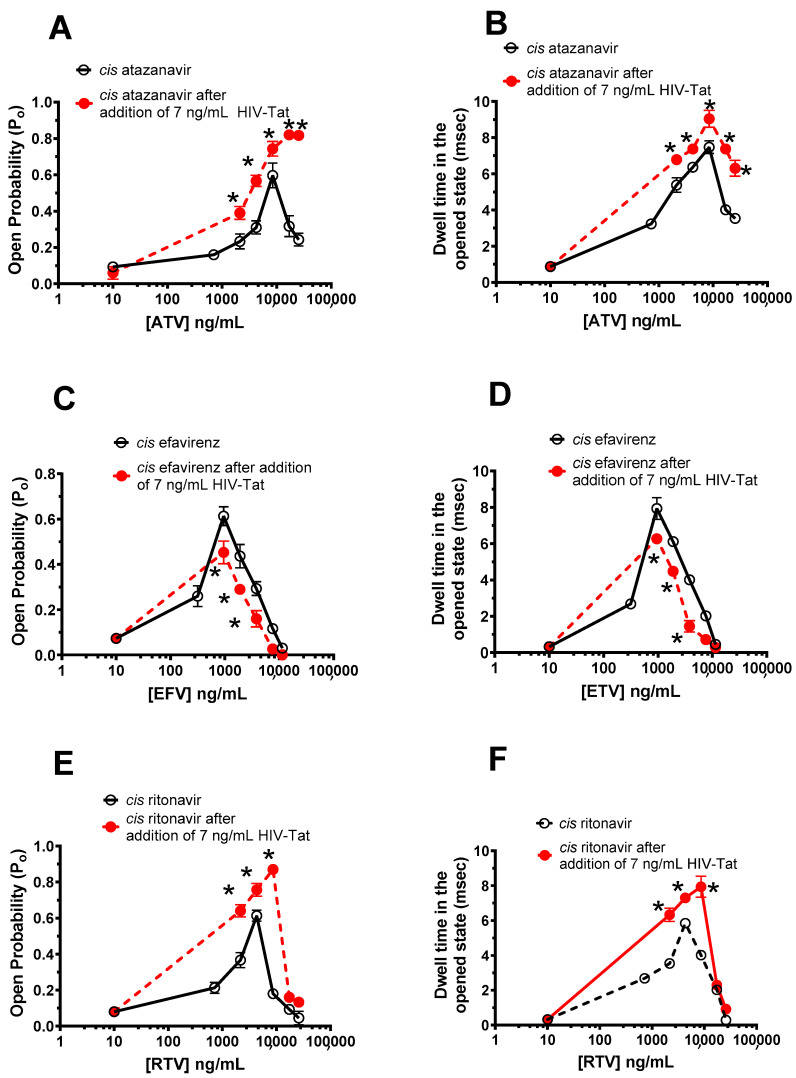
The effects of varying *cis* concentrations of atazanavir (ATV), efavirenz (EFV), and ritonavir (RTV) on the P_o_ and dwell time in the opened state of RyR2 in the presence and absence of low HIV-Tat (14 ng/mL). **Panels A**,**B** show graphs of the P_o_ and dwell times in the opened state of RyR2 as a function of varying concentrations of *cis* ATV in the absence (○) and presence (●) of 7 ng/mL HIV-Tat in the *cis* chamber. Data shown are mean ± S.E.M for n = 13 channels. **Panels C**,**D** show graphs of the P_o_ and dwell times in the opened state of RyR2 as a function of varying concentrations of *cis* EFV in the absence (○) and presence (●) of 7 ng/mL HIV-Tat in the *cis* chamber. Data shown are mean ± S.E.M for n = 112 channels. **Panels E**,**F** show graphs of the P_o_ and dwell times in the opened state of RyR2 as a function of varying concentrations of *cis* RTV in the absence (○) and presence (●) of 7 ng/mL HIV-Tat in the *cis* chamber. Data shown are mean ± S.E.M for n = 11 channels. * denotes significant differences (*p* < 0.05) from that in the absence of HIV-Tat.

Experiments were conducted to investigate if pretreating RyR2 with a low HIV-Tat (7 ng/mL), which exhibited a small effect on the P_o_ of RyR2, would synergize and/or antagonize the actions of ATV, EFV, and RTV on RyR2. We found that when a RyR2 with 1.0 μM *cis* Ca^2+^ was pretreated with 7 ng/mL HIV-Tat (*cis*), the ability of ATV and RTV to activate RyR2 was enhanced (Figure 8A,E; see also raw data in Supplemental Figures S2 and S4). The exaggerated activation of RyR2 by ATV and RTV arose from an increase in the dwell time in the opened state (Figure 8B,F). When RyR2 in 1.0 μM *cis* Ca^2+^ was pretreated with 7 ng/mL HIV-Tat, the ability of EFV to activate RyR2 was blunted and the closure of RyR2 by EFV was enhanced, primarily by decreasing the dwell time in the open state and the gating frequency (Figure 8C,D; see also raw data in Supplemental Figure S3).

### 2.6. In Silico Molecular Docking of HIV-Tat on RyR2

**Figure 9:**
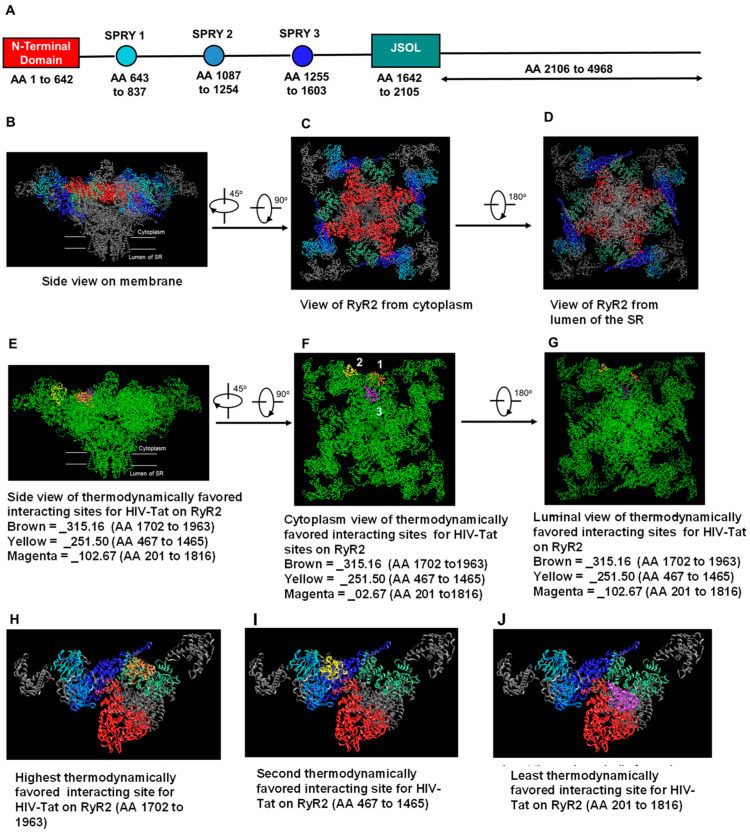
Locations of the thermodynamically favored interaction sites for HIV-Tat on RyR2. **Panel A** shows the overall structure of the closed (with the N-terminal) domain: SPRY1, SPRY2, and SPRY 3, and the JSOL domain. **Panels B**–**D** show the tetrameric structure of RyR2 at 4.2 Å resolution, which is domain-colored with the same scheme as in (**A**), with a view from the membrane, from the cytoplasm, and a view from the lumen of the SR, respectively. **Panels E**–**G** show the three thermodynamically favored docking sites for HIV-Tat on RyR2, with a view from membrane, from the cytoplasm, and a view from the lumen of the SR, respectively. The numbers in **Panel F** refer to the highest (1) to lowest (3) thermodynamically favored sites. **Panels H**–**J** show the three thermodynamically favored docking sites for HIV-Tat on a RyR2 monomer in relation to the N-terminal domain: SPRY1, SPRY2, and SPRY 3, and the JSOL domain, which are color-coded to that within Figure 9A.

Having established that HIV-Tat binds to and activates RyR2, and that pretreating RyR2 with a low HIV-Tat (7 ng/mL) exacerbated the actions of ATV, EFV, and RTV, in silico molecular docking studies were then conducted using Schrodinger’s Prime protein–protein docking software to identify the thermodynamically favored interacting sites for HIV-Tat on a reconstructed human RyR2. Figure 9A–C show 3D reconstruction images of human RyR2 projected onto the Protein Data Bank structures of Sus scrofa RyR2 in the opened state (5GOA) at 4.2 Å, as viewed from the membrane, from the cytoplasm, and from inside the lumen of the SR. The N-terminal domain: SPRY1, SPRY2, SPRY3, and the JSOL (or hinge domain) are shown in Figure 9A and are color-coded to easily view these sites on the 3D structure of RyR2 [52,53,54]. After mixing HIV-Tat with RyR2 in silico and performing 70,000 rotations and energy evaluations, three thermodynamically favored interaction sites for HIV-Tat on RyR2 were identified (Figure 8E–G, showing views from the membrane, cytoplasm, and lumen of the SR, respectively). Site 1 (yellow) was the most thermodynamically favored, with a binding energy of −315.16 AU; site 2 (brown) had a binding energy of −251.50 AU, and site 3 (magenta) had a binding energy of −102.67 AU. When using Schrodinger’s Prime protein–protein docking software, the more negative the binding energy, the better the fit. The most thermodynamically favored site of interaction (site 1: brown) for HIV-Tat resides in the region within the junctional solenoid region (JSOL: amino acids (AA) 1702–1963) [56,57], with 14 hydrogen bonding, 3 salt bridges, and 1 pi stack (See Table 1). Note, the numbering of the AA for HIV-Tat that is listed is derived from the crystal structure provided by Protein Data Bank (PDB id; 1TIV). The second thermodynamically favored site of interaction (site 2: yellow) resides within the SPRY1 (AA 647–659), with 2 hydrogen bonding and 2 salt bridges, the SPRY2 domain (AA 1151–1249), with 5 hydrogen bonding, and the SPRY 3 domain (AA 1335–1465), with 5 hydrogen bonding and 1 salt bridge (Table 2). The least thermodynamically favored of the three sites (site 3: magenta) interacts with the N-terminal domain of RyR2 between AA 201 and 554, with 13 hydrogen bonding and 1 pi stack, and the JSOL domain between AA 1739–1816, with 6 hydrogen bonding and 2 salt bridges (Table 3).

## 3. Discussion

PLWH, especially those with inadequate viral suppression and a low CD4^+^ T-cell count (<200 cells/mm^3^), are 2.5–4 times more likely to succumb to SCD compared to uninfected individuals [2,3,4,5,10]. The available data suggest nonarrhythmic and arrhythmic causes for SCD, with the former attributed to occult drug overdose [10]. To date, the arrhythmic causes of SCD are thought to arise principally from the prolongation of the QT interval by HIV proteins, select antiretroviral and prescription medications, recreational and illicit drugs, and from myocardial fibrosis.

Here, we identify another potential mechanism that could be increasing the risk of arrhythmias and SCD, i.e., the disruption of the opening and closing of RyR2 by HIV-Tat and select antiretroviral drugs. This conclusion is based on several novel findings. First, we show that HIV-Tat, ATV, and RTV enhanced equilibrium [^3^H]ryanodine binding to RyR2 and that EFV attenuated equilibrium [^3^H]ryanodine binding to RyR2. The other antiretroviral drugs investigated, ABC, BIC, TDF, and RPV, had no measurable effects on equilibrium [^3^H]ryanodine binding to RyR2. In this study, we also show, for the first time, that when RyR2 was preincubated with HIV-Tat (reminiscent of inadequate viral suppression), the abilities of ATV, EFV, and RTV to modulate equilibrium [^3^H]ryanodine binding to RyR2 were enhanced as indicated by leftward shifts in their binding curves. However, since SR membrane vesicles were used, it was not clear whether the abilities of HIV-Tat, ATV, EFV, and RTV to alter equilibrium [^3^H]ryanodine binding arose from direct interactions with RyR2 or from their interactions with endogenous proteins that are known for their RyR2 activity.

Compounds that alter equilibrium [^3^H]ryanodine binding to RyR2 in SR vesicles should also modulate the activity of RyR2 in vivo. As such, we investigated whether HIV-Tat, ATV, EFV, and RTV can perturb intracellular Ca^2+^ homeostasis in cardiac myocytes. For this, freshly isolated primary rat ventricular myocytes were loaded with the Ca^2+^-sensitive dye Fluo-3, and live-cell imaging was conducted before and after exposure to these agents. In these studies, we found that HIV-Tat, ATV, EFV, and RTV elicited Ca^2+^ transients and/or waves in myocytes within one minute (after exposure). However, the Ca^2+^ transient/wave patterns were distinct for each compound, with ATV eliciting repeated Ca^2+^ waves and EFV and HIV-Tat eliciting less frequent but larger global Ca^2+^transients. RTV also triggers Ca^2+^ release, firstly, at multiple distinct regions within the myocyte, which then transitions to short-lasting global Ca^2+^ elevation. Persistent Ca^2+^ release from the SR can activate Ca^2+^-sensitive signaling pathways, which may disrupt cellular functions. The increases in intracellular Ca^2+^ elicited by HIV-Tat, ATV, EFV, and RTV were also blunted when the myocytes were pretreated with ryanodine to close RyR2. Although these data are consistent with the notion that HIV-Tat, ATV, EFV, and RTV modulate RyR2, it was still not clear whether their effects on myocyte Ca^2+^ homeostasis arose from their direct interactions with RyR2 or from their interactions with the endogenous regulatory modulators of RyR2.

In order to address this question, lipid bilayer single-channel studies were conducted. For this, the junctional vesicles were solubilized with the mild detergent CHAPS to remove the attached RyR2-associated/modulatory protein. Purified RyR2 that was reconstituted into proteoliposomes was then fused to artificial lipid membranes, and single-channel assays were conducted. Using this approach, we show for the first time that HIV-Tat, ATV, EFV, and RTV dose-dependently increase the P_o_ of RyR2, primarily by increasing its dwell time in the open state. The effects of HIV-Tat were not dependent on trans Ca^2+^ levels, i.e., the Ca^2+^ content inside the SR. These data provide the first direct evidence that HIV-Tat, ATV, EFV, and RTV interact directly with the RyR2 isolated from bovine hearts. Because of the high degree of sequence homology among species, we expect HIV-Tat, ATV, EFV, and RTV will also bind directly to and modulate RyR2 from other species.

In this study, we also found that HIV-Tat at concentrations 26 and 42 ng/mL also induced a reversible state of reduced conductance that was 50% of the maximum opening. This state of reduced conductance (R1) was not seen with lower or higher concentrations of HIV-Tat. At present, the specific molecular reasons for the induction of a reversible state of reduced conductance by 28 and 42 ng/mL HIV-Tat are not known. In an attempt to answer this question, a in silico molecular docking study was conducted to identify the thermodynamically favored regions on RyR2 where HIV-Tat could be binding. Using Schrodinger’s Prime protein–protein docking software, three thermodynamically favored sites of interaction for HIV-Tat on RyR2 were identified. The highest interacting site for HIV-Tat on RyR2 was mapped to the junctional solenoid region (JSOL: AA 1702–1963). Other thermodynamically favored sites of interaction reside in the SPRY domains, the N-terminal domain, and the JSOL domain [56,57]. All these sites are critical for stabilizing the RyR2 monomers and for regulating the gating/conductance of RyR2. The JSOL region, equivalent to the “handle domain” [56], is involved in intra- and intersubunit interactions [57], and the destabilization of these interactions could alter the activity and conductance of RyR2. The SPRY domains are involved in fine-tuning the activity of RyR2 [57,58]. The NTD interacts with itself to promote channel closure, as well as to support the formation of functional tetrameric RyR2 channels. Immunophilin FKBP12.6, for which the binding site has been mapped to AA 305–1937, helps prevent Ca^2+^ leaking from RyR2 [59,60,61], and its dissociation by HIV-Tat could have deleterious clinical consequences, including SCD. In an earlier report, Kaftan et al. [62] showed that when rapamycin was used to deplete FKBP12.6 from RyR2, the conductance of RyR2 was reduced. Jayaraman et al. [63] also showed that solubilization with detergent was not sufficient to remove immunophilins from ryanodine receptors. Since our “purified” RyR2 contained FKBP12.6 (see Supplemental Figure S5) and a thermodynamically favored docking site for HIV-Tat that resides within the binding site for FKBP12.6 (AA 305–1937 on RyR2), [60,61], it stands to reason that HIV-Tat could be reversibly binding to FKBP12.6. However, this hypothesis must be experimentally tested.

Here, we also show for the first time that pretreating RyR2 with a dose of HIV-Tat that does not significantly alter the P_o_ of RyR2 exacerbated the ability of ATV and RTV to activate RyR2 and EFV to close RyR2. These findings are of significant importance and need to be expanded upon for multiple reasons. First, activating RyR2 could deplete the Ca^2+^ content inside the SR, which may reduce the force of myocardial contractions. “Leaky” RyR2 can also trigger myocyte contractions independent of membrane depolarization. When the latter occurs, the Na^+^–Ca^2+^ exchanger (NCX) on the plasma membrane will extrude the intracellular Ca^2+^ in exchange for an influx of Na^+^, resulting in delayed after depolarizations (DADs), aberrant myocyte contractions, and arrhythmias, some of which may be fatal [37,64]. A sudden tachycardia by common stressors that activate the sympathetic nervous system can also trigger arrhythmias. Common stressors include physical exercise, emotional stress, and recreational and illicit drugs [65]. Second, when RyR2 is unable to be sufficiently activated by the Ca^2+^ that enters the cell following depolarization, the amplitude of Ca^2+^ released from the SR will be reduced, and the force of the myocardial contractions will also be reduced. Since a smaller amount of Ca^2+^ is released from the SR following depolarization, the repeated refilling of the SR via SERCA could increase the Ca^2+^ content inside the SR. At some maximal SR Ca^2+^ load, depolarization could result in a decreased but sustained release of Ca^2+^ from the SR. A prolonged rise in intracellular Ca^2+^ will supercharge the electrogenic NCX, generating a depolarizing inward Na^+^ current as Ca^2+^ is extruded. This aberrant depolarization, known as early after depolarization (EAD), can serve as a trigger an arrhythmia, a precursor for SCD [45,66,67]. Third, animal models to assess the arrhythmia risks posed by ATV, EFV, RTV, and other antiretroviral agents should include, at a minimum, HIV-Tat. Whether other HIV-1 auxiliary proteins (gp120 Nef) and inflammatory cytokines are also needed remains to be evaluated. Without these components in animal models, it would be challenging to decipher why PLWH, especially those with inadequate viral suppression, are at significant risk of SCD [3].

This study is not without limitations. First, only a limited number of available antiretroviral agents and HIV-Tats were tested. Of the seven antiretroviral drugs tested, only ATV, EFV, and RTV bind to and modulate RyR2; the others did not. Although these drugs are not generally used as first-line drugs in developed economies, they continue to be used in developing countries where the incidence of HIV-1 infection is high [48]. EFV at a dose of 400 mg in combination with an NRTI is recommended as the alternative first-line regimen for antiretroviral naïve adults and adolescents living with HIV who are initiating ART treatment. ATV is an alternate second-line protease inhibitor used in combination with ritonavir to increase efficacy [48]. Second, although we show that HIV-Tat, ATV, EFV, and RTV can elicit Ca^2+^ waves and global Ca^2+^ transients in primary rat cardiac myocytes, we have not specifically demonstrated the development of arrhythmia and SCD using animal models. Third, the in silico mutation studies that identify the potential docking sites of HIV-Tat to RyR2 is the first step. These interacting sites must be validated using mutation studies in silico and in cell culture models.

In summary, the present study shows for the first time that low HIV-Tat and therapeutic levels of ATV, EFV, and RTV can bind to and modulate the function of RyR2. ABC, BIC, RPV, and TDF had no significant effect on the equilibrium binding of [^3^H]ryanodine to RyR2. In the presence of low HIV-Tat (analogous to inadequate HIV-1 viremia control), the abilities of ATV and RTV to activate RyR2 were potentiated, while the ability of EFV to close RyR2 was enhanced. We also show that HIV-Tat, ATV, EFV, and RTV can perturb intracellular Ca^2+^ homeostasis in primary rat ventricular myocytes via a RyR2-sensitive mechanism and that there are potential interacting sites for HIV-Tat on RyR2. Although these data indicate that HIV-Tat, ATV, EFV, and RTV can modulate RyR2 in cardiac myocytes, additional studies in clinically relevant models must be conducted to determine if these drugs are capable of triggering arrhythmias and even SCD.

## 4. Materials and Methods

### 4.1. Antibodies, Reagents, and Drugs

[^3^H]Ryanodine was purchased from Perkin-Elmer Health Science Inc (Boston, MA, USA). Phosphatidylserine, phosphatidylcholine, and phosphatidylethanolamine were obtained from Avanti Polar Lipids Inc. (Alabaster, AL, USA). Free-base efavirenz (EFV) and ritonavir (RTV) were obtained from Shengda Pharmaceutical Co (Zhejiang, China), and atazanavir (ATV) sulfate was purchased from Gyma Laboratories of America, Inc (Westbury, NY, USA). Abacavir (ABC), bictegravir (BIC), rilpivirine (RPV), and tenofovir disoproxil fumarate were purchased from Boc Sciences (Shirley, NY, USA). Dialysis membranes were obtained from Spectrum Laboratories Inc (Rancho Dominguez, CA, USA). Recombinant HIV-Tat protein (ab83353) was obtained from Abcam (Waltham, MA, USA). All other reagents and solvents used were of the highest grade commercially available.

### 4.2. Preparation of Stock Solutions of HIV-Tat, EFV, ATV and RTV

HIV-Tat was prepared by dissolving 100 µg in 100 µL of apirogenic sterile water, and stock solution was aliquoted and stored at −80 °C in silanized vials until use. ATV (17 mg), EFV (8 mg), and RTV (14 mg) were dissolved in 2.5 mL of ethanol and the suspensions were vortexed for 5 min at room temperature (22 °C) and centrifuged at 20,000× *g* for 30 min. The supernatants were removed and diluted 10× with methanol, and high-performance liquid chromatography (using a YMC Octyl C8 column, Waters Inc., Milford, MA) with a mobile phase consisting of 52% 25 mM KH_2_PO_4_, pH 4.15/48% acetonitrile at a flow rate of 0.4 mL/min and a UV/V is detector at 212 nm) was used to determine the concentrations of the drugs in solution [50]. ABC (14.31 mg), BCT (22.46 mg), RPV (14.36 mg), and TDF (18.32 mg) we dissolved in dimethyl sulfoxide (DMSO) to generate 50 mM stock solutions. These drugs were further diluted 1:10 in DMSO for working solutions and 1:100 for binding assays.

### 4.3. Preparation SR Vesicles and Enrichment of RyR2 from Bovine Hearts

Bovine hearts were generously donated by the Greater Omaha Packaging Company (Omaha, NE, USA).

*(a) Sarcoplasmic reticulum vesicles:* Sarcoplasmic reticulum (SR) membrane vesicles were prepared using the procedure described earlier [53,68]. Briefly, after removal of outer fat, ventricular tissues were cut into small pieces, placed in ice-cold isolation buffer (10 mM NaHCO_3_, 230 μM phenylmethylsulfonyl fluoride and 1.1 μM leupeptin, pH 7.4) and homogenized for 3 × 30 s using a Kinematica PT-600 Polytron at setting 4.5. Homogenates were then placed in Beckman type JA- 10 centrifuge tubes (250 mL) and centrifuged for 20 min at 7500× *g*_av_. The pellets were collected and rehomogenized (3 × 30 s interval) in isolation buffer at a setting of 6.0. Homogenates were centrifuged at 11,000× *g*_av_ for 20 min, and supernatants were filtered through four layers of cheese cloth, and microsomal vesicles were obtained by sedimentation at 85,000× *g*_av_ for 30 min at 4 °C in a Beckman type 45Ti rotor. The pellets containing the microsomal vesicles were resuspended in buffer (0.3 M sucrose, 10 mM histidine, 230 μM PMSF, and 1.1 μM leupeptin, pH 7.4), flash frozen, and stored at −80 °C.

*(b) Junctional SR vesicles:* discontinuous sucrose gradient centrifugation was used to separate junctional SR vesicles from microsomal vesicles, as described in [53,69]. For this, microsomal membranes (5 mL) were layered onto centrifuge tubes containing discontinuous sucrose gradients (bottom to top: 5 mL of 1.5 M, 7 mL of 1.2 M, 7 mL of 1.0 M, and 7 mL of 0.8 M sucrose in 30 mL tubes). The tubes were then centrifuged in a Beckman SW-28 swinging bucket-type rotor at 110,000× *g*_av_ for 2 h at 4 °C. The membrane fractions that sedimented at the 1.2 M/1.5 M sucrose interface were collected and resuspended in 10 mM histidine, 230 μM PMSF, and 1.1 μM leupeptin, and the junctional SR vesicles were recovered by sedimentation at 100,000× *g*_av_ for 30 min at 4 °C. The pellet was resuspended in 0.3 M sucrose, 10 mM histidine, 230 μM PMSF, and 1.1 μM leupeptin, flash frozen, and stored at −80 °C.

*(c) Proteoliposomes* containing RyR2 were prepared as described earlier [68,69]. For this, junctional SR vesicles were solubilized in buffer (1.0 M NaCl, 0.05 mM EGTA, 0.35 mM Ca^2+^, 5 mM AMP, 20 mM Na/PIPES, pH 7.4, 0.3 mM Pefabloc, 0.03 mM leupeptin, 0.9 mM dithiothreitol, and 5 mg/mL phosphatidylcholine) containing 1.5% CHAPS on ice for 10 min and then centrifuged at 26,000× *g*_av_ for 30 min. The supernatant was then layered onto a 7–15% linear sucrose gradient (3 mL of solubilized protein onto 34 mL per gradient, 6 tubes) and centrifuged at 89,500× *g*_av_ (Beckman Ultra Centrifuge, SW28 rotor) for 17 h at 4 °C. [^3^H]ryanodine (3.3 nM) was mixed into the sample of one tube before centrifugation for locating RyR2. After centrifugation, 2 mL aliquots were collected from the bottom of tube and the location of RyR2 was identified by [^3^H]ryanodine binding (scintillation counter). SDS-polyacrylamide gel electrophoresis (4–15%) of aliquots, followed by silver staining, was used to verify RyR2 protein. Fractions enriched in RyR2 protein were pooled and dialyzed at 4 °C for 44 h in a buffer containing 0.5 M NaCl, 0.1 mM EGTA, 0.2 mM CaCl_2_, 10 mM Na/PIPES, pH 7.4, 0.1 mM DTT, and 0.1 mM PMSF, with three changes. Proteoliposomes were then centrifuged at 163,500× *g*_av_ for 2 h at 4 °C, and the pellet was then resuspended in buffer containing 10 mM Na/PIPES, pH 7.4, 0.1 mM DTT and 0.3 M sucrose, and 100 μL aliquots were flash-frozen and stored in the vapor phase of liquid nitrogen.

### 4.4. Ability of HIV-Tat, ATV, EFV, and RTV to Bind to Modulate RyR2

*(a) Equilibrium [^3^H]ryanodine displacement/potentiation assays:* SR membrane vesicles (0.1 mg/mL) were incubated in buffer (500 mM KCl, 20 mM Tris·HCl, 0.03 mM Ca^2+^, pH 7.4) with 6.7 nM [^3^H]ryanodine and varying concentrations of HIV-Tat (0–56 ng/mL), ABC (0–14,315 ng/mL), ATV (0–21,120 ng/mL), BCT (0–22,469 ng/mL), EFV (0–18,900ng/mL), RPV (0–14,360 ng/mL), RTV (1–21,600 ng/mL), TDF (0–18,321 ng/mL) or ryanodine (0 to 147 ng/mL) for 2 h at 37 °C [50,53]. Nonspecific equilibrium [^3^H]ryanodine binding was determined by incubating a separate set of vesicles with 1 µM (493.5 ng/mL) unlabeled ryanodine. After incubation, vesicles were vacuumed filtered through GFB filter paper, washed with ice-cold buffer, and the [^3^H]ryanodine bound to the filter paper was determined by liquid scintillation counting. Data were analyzed using nonlinear regression analyses fitted to one- and two-site competition models using GraphPad Prism (Version 9).

*(b) Effect of HIV-Tat pretreatment on the ability of ATV, EFV, and RTV to displace/potentiate [^3^H]ryanodine from RyR2:* SR membrane vesicles (0.1 mg/mL) in binding buffer (500 mM KCl, 20 mM Tris·HCl, 0.03 mM Ca^2+^, 2 mM reduced glutathione, and 100 μM EGTA, 6.7 nM [^3^H]ryanodine, pH 7.4) were incubated with HIV-Tat (14 ng/mL) for 10 min at 37 °C. After this, varying amounts of ATV (0–21,120 ng/mL), EFV (0–18,900ng/mL), and RTV (1–21,600 ng/mL) were added, and incubation continued for 2 h at 37 °C. Nonspecific [^3^H]ryanodine binding was determined by incubating vesicles with 1 µM (493 ng/mL) unlabeled ryanodine. After incubation, vesicles were vacuumed filtered, washed with ice-cold buffer, and the [^3^H]ryanodine bound to the filter paper was determined by liquid scintillation counting. Data were analyzed using nonlinear regression analyses fitted to one- and two-site competition models using GraphPad Prism (Version 9).

*(c) Single channel recordings:* A bilayer cup with a 250 µm diameter hole was placed in a cup holder, and each side was filled with 0.25 mM KCl and 20 mM K-HEPES, pH 7.4. [50,68,69]. A mixture of phosphatidylethanolamine, phosphatidylserine, and phosphatidylcholine at a ratio of 5:3:2 (35 mg/mL of lipid) in *n*-decane was then used to paint a lipid bilayer across the hole of the cup to separate the two chambers. The calcium (1.0 µM) and proteoliposomes were then added to one chamber labeled *cis* (equivalent to the cytoplasm), and a single RyR2 channel was allowed to fuse into the bilayer. The other chamber of the bilayer was designated as the *trans* side (equivalent to the lumen of the SR) and served as ground.

*(i)*  *Effects of ATV, EFV, RTV, and HIV-Tat on the gating and conductance of RyR2*. ATV (0–25,344 ng/mL), EFV (0–11,376 ng/mL), RTV (0–25,956 ng/mL), or HIV-Tat (7–52 ng/mL) was cumulatively added to the *cis* chamber of the bilayer (cytoplasmic side of RyR2). After each dose, the chamber was vigorously stirred for 15 s, and three 2-min recordings were obtained at + 35 mV holding potential. All recordings were carried out at room temperature (23–25 °C). Data acquisition was performed using commercially available instruments and software (Axopatch 1D, Digidata 1322A and pClamp 10.0, Axon Instruments, Burlingame, CA, USA). Electrical signals were filtered at 2 kHz, digitized at 10 kHz, and analyzed as described previously [68,69]. Data were obtained using pClamp 10.0 (Axon Instruments, Burlingame, CA, USA) and GraphPrism 7.0 (La Jolla, CA, USA).
*(ii)*  *Effects of HIV-Tat pretreatment on the ATV, EFV, and RTV effects on RyR2*. HIV-Tat (7 ng/mL) was added to the *cis* chamber after a single RyR2 was incorporated into the bilayer. After vigorously stirring for 15 s, three 2-min-long recordings at +35 mV were used to obtain activity. Thereafter, ATV (0–25,344 ng/mL), EFV (0–11,376 ng/mL), or RTV (0–25,956 ng/mL) was added to the *cis* chamber in cumulative amounts. After the addition of each dose of the antiretroviral agent, the *cis* chamber was vigorously stirred for 15 s, and three 2-min-long recordings at +35 mV were obtained.

### 4.5. Effects of HIV-Tat, ATV, EFV, and RTV on Intracellular Ca^2+^ Homeostasis in Primary Rat Ventricular Myocytes

Male Sprague-Dawley rats (300 g) were obtained from Charles River Laboratories (New York, NY, USA) with approval from Institutional Animal Care and Use. Isolation of rat ventricular myocytes is detailed in our earlier work [70]. After isolation. myocytes in Dubelcco’s Modified Eagle Medium containing 1.8 mM Ca^2+^, supplemented with F-12 (DMEM-F12) and antibiotics (100 U/mL penicillin, 100 μg/mL streptomycin, and 100 μg/mL gentamicin), pH 7.3, were incubated for 1 h at 37 °C in Petri dishes containing glass cover slips that had been previously coated with laminin. After incubation, unbound myocytes were gently removed by suction, and DMEM-F12 was replaced with Tyrode solution. Myocytes were then loaded with fluo-3 (5 μM) for 30 min at 37 °C, washed, and placed on the head stage of a laser confocal microscope equipped with 10× and 40× objectives (Zeiss Confocal LSM 710, equipped with an Argon-Krypton Laser, 25 mW argon laser, 2% intensity, Thornwood, NJ, excitation wavelength 488 nm, and emission wavelengths of 515 nm). HIV-Tat (25 ng/mL), ATV (5.0 μM), EFV (5.0 μM), and RTV (5.0 μM) were manually added to a corner of the glass chamber, and images were recorded every 1 s for up to 240 min. Experiments were repeated with a second set of cells that were pre-treated with ryanodine (50 μM) for 10 min prior to the addition of the HIV-Tat (25 ng/mL), ATV (5.0 μM), EFV (5.0 μM), and RTV (5.0 μM). Data were analyzed using LSM Meta 5.0, Microsoft Excel (Microsoft, Seattle, WA, USA), and Prism (GraphPad, Version 9, La Jolla, CA, USA).

### 4.6. In Silico Molecular Docking of HIV-Tat on RyR2

The sequence of the human RyR2 receptor was obtained from Uniprot: accession code Q92736. The template sequence was projected onto the Protein Data Bank structures of Sus scrofa RyR2, representing the closed (PDB id: 5GO9) and open (5GOA) form. Gaps in the structural data were filled with loops modeled from Sus scrofa and Oryctolagus cuniculus according to best PSI-BLAST score. HIV-Tat protein was also obtained from Protein Data Bank (PDB id; 1TIV). The Schrodinger 2022-1 program package was used for all calculations and analyses. The monomeric model of RyR2 was then subjected to a restrained minimization with the OPLS4 force field. Schrodinger Prime was used to reconstruct the final tetrameric model for RyR2 at the same 4.2 Å resolution [52].

Schrodinger Prime protein–protein docking was then used to dock the HIV-Tat protein to the RyR2 monomer. A total of 30 poses representing the best of 70,000 rotations and energy evaluations for each pose were obtained. Poses that did not interact with the cytosolic cap were not considered for further analysis. The remaining poses were ranked by Prime docking score, and the three with the highest interactions were selected.

### 4.7. Statistical Analysis

Paired student *t*-tests were used to compare data before and after drug treatment using Microsoft Excel (Microsoft Corporation, Seattle, WA, USA). One-way analysis of variance (ANOVA), followed by the Bonferroni’s post hoc test, were also used for some analyses using GraphPad Prism 7.0 (La Jolla, CA, USA). Data are presented in text, and graphs as the mean standard error of mean (S.E.M). Significance was determined at the 95% confidence interval.

## Figures and Tables

**Table 1:** Thermodynamically favored interactions between RyR2 and HIV-Tat at site 1.

RyR2 Residues	HIV-Tat Residues	Distance (A)	Specific Interactions
A:Lys 1963	B:Asp 279	2.3	1× hydrogen bonding (hb)
A:Arg 1960	B:Asp 279	2.3	1× salt bridge
A:His 1920	B:Glh 278	1.9	1× hb
A:Arg 1919	B:Asp 279	2.3	1× hb
A:Asp 1915	B:Arg 340	2.2	2× hb, 1× salt bridge
A:Tyr 1912	B:Gln 341	1.8	1× hb
A:Ser 1869	B:Ash 312	1.8	1× hb
A:Tyr 1826	B:Phe 277	2.4	1× hb
A:Ile 1822	B:Arg 309	2.2	1× hb
A:Phe 1782	B:Phe 307	2.7	1× pi stack
A:Arg 1758	B:Pro 281	2.2	1× hb
A:Lys 1744	B:Asp 352	2.3	1× hb, 1× salt bridge
A:Lys 1744	B:Arg 261	2.2	1× hb
A:Asn 1743	B:Asp 352	1.8	1× hb
A:Tyr 1702	B:Arg 264	2.2	1× hb

**Table 2:** Thermodynamically favored Interactions between RyR2 and HIV-Tat at site 2.

RyR2 Residues	HIV-Tat Residues	Distance (A)	Specific Interactions
A:Val 1465	B:Arg 309	2.0	1× hydrogen bonding (hb)
A:Arg 1463	B:Glh 278	2.2	1× hb
A:Arg 1383	B:Asp 279	2.1	1× hb
A:Lys 1380	B:Asp 308	2.3	1× hb, 1× salt bridge
A:Glu 1335	B:Asn 285	2.1	1× hb
A:Met 1249	B:Arg 292	1.9	1× hb
A:Ile 1247	B:Gln 300	2.4	1× hb
A:Asp 1246	B:Gln 300	2.5	1× hb
A:Val 1161	B:Arg 302	2.2	2× hb
A:Ile 659	B:Lys 297	2.4	1× hb
A:Arg 647	B:Glu 286	2.2	1× hb, 1× salt bridge
A:Arg 647	B:Asp 280	2.9	1× salt bridge

**Table 3:** Thermodynamically favored Interactions between RyR2 and HIV-Tat at site 3.

RyR2 Residues	HIV-Tat Residues	Distance (A)	Specific Interactions
A:Phe 1816	B:Arg 302	1.7	2× hydrogen bonding (hb)
A:Glu 1815	B:Arg 292	2.4	1× hb, 1× salt bridge
A:Ser 1779	B:Arg 292	2.2	1× hb
A:Tyr 1776	B:Glu 289	2.0	1× hb
A:Lys 1744	B:Asp 308	2.5	1× salt bridge
A:Phe 1739	B:Leu 282	2.0	1× hb
A:Ser 554	B:Ash 279	1.6	2× hb
A:Ser 554	B:Glu 278	2.1	1× hb
A:Arg 520	B:Asp 276	2.2	1× hb
A:Phe 514	B:Glu 278	1.8	1× hb
A:Arg 480	B:Gly 338	1.8	1× hb
A:Arg 480	B:Met 271	2.2	1× hb
A:Gln 476	B:Glu 286	2.3	1× hb
A:Phe 429	B:Trp 336	2.4	1× pi stack
A:Ser 326	B:Lys 297	1.8	1× hb
A:Ser 326	B:Tyr 327	1.8	1× hb
A:Lys 325	B:Gln 300	2.1	1× hb
A:Trp 212	B:Tyr 327	2.0	1× hb
A:Leu 201	B:Arg 335	2.2	1× hb

## Data Availability

The datasets generated for this study are available from the corresponding author upon request.

## Supplementary Materials

The supporting information can be downloaded at: https://www.mdpi.com/article/10.3390/ijms24010274/s1.
