# Supplementary material for: HIV-Tat Exacerbates the Actions of Atazanavir, Efavirenz, and Ritonavir on Cardiac Ryanodine Receptor (RyR2)
Source: Int J Mol Sci. 2022 Dec 23;24(1):274. doi: 10.3390/ijms24010274 (PMC9820108; doi:10.3390/ijms24010274)
Supplement: Supplementary file 1 [file ijms-24-00274-s001.zip › Supplemental information -IJMS 984856, 12-19- 2022.pdf]

Supplemental Information

HIV-Tat Exacerbates the actions of Atazanavir, Efavirenz and Ritonavir on cardiac ryanodine receptor (RyR2)

Fadhel A. Alomar[1*], Chengju Tian[2*], Sean R. Bidasee[2], Zachary Venn[2], Evan Schroder[2], Nicholas Polermo[3], Mohammad Alshabeeb[4], Benson J. Edagwa[2], Jason Payne[5], and Keshore R. Bidasee[2,6,7]

[1]Department of Pharmacology and Toxicology, College of Clinical Pharmacy, Imam Abdulrahman Bin Faisal University, Dammam 31441, Saudi Arabia, [2]Departments of Pharmacology and Experimental Neuroscience, University of Nebraska Medical Center, Omaha, NE 68198, [3]Department of Pharmaceutical Sciences, University of Nebraska Medical Center, Omaha NE, [4]Population Health Research Section, King Abdullah International Medical Research Center, King Saudi bin Abdulaziz University for Health Sciences, Riyadh, Saudi Arabia, [5]Department of Internal Medicine, University of Nebraska Medical Center, Omaha, NE 68198, and [6]Department of Environment and Occupational Health, University of Nebraska Medical Center, Omaha, NE 68198, and [7]Nebraska Redox Biology Center, Lincoln NE.

Corresponding Author:

Keshore R. Bidasee, Ph.D.,

Department of Pharmacology and Experimental Neuroscience, 985800 Nebraska Medical Center, Durham Research Center, DRC 3047, Omaha, NE, USA. Tel: (402) 559-9018; Fax: (402) 559-7495, E-mail: kbidasee@unmc.edu.

Effects of heat-inactivated HIV-Tat on RyR2

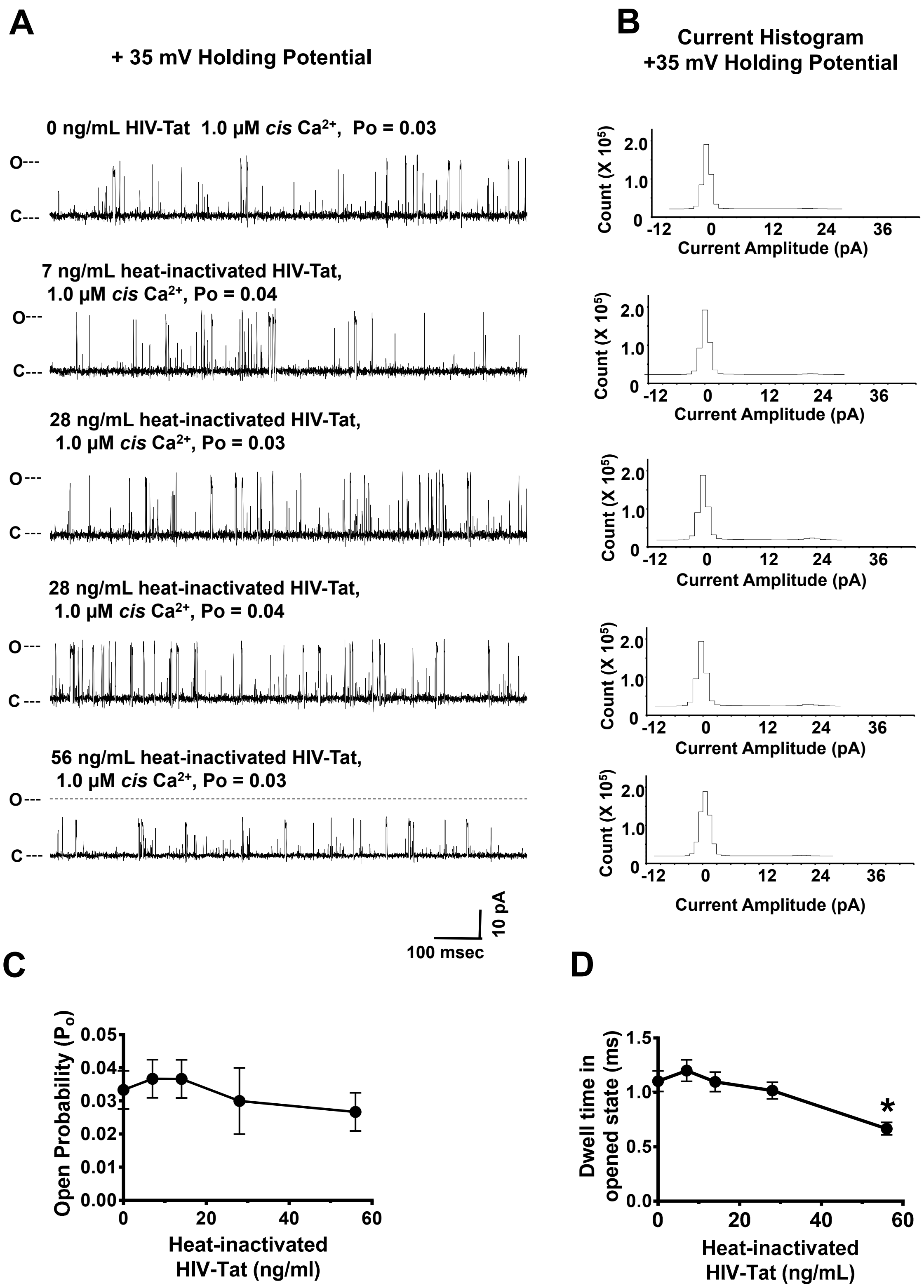

Supplemental Figure S1: Effects of heat-inactivated HIV-Tat on the open probability of RyR2. **Panel A** shows a representative 1.0 sec recording of a RyR2 channel with 1.0 µM *cis* Ca^{2+} and cumulative amounts of *cis* heat-inactivated HIV-Tat. **Panel B** shows current histograms for that channel with cumulative amounts of *cis* heat-inactivated HIV-Tat. **Panels C and D** are graphs of the mean open probability and dwell times of RyR2 as a function of *cis* concentrations of HIV-Tat channels for n = 8 channels. Recordings shown are at +35 mV (upward deflections) in symmetric KCl buffer solution (0.25 mM KCl, 20 mM K/HEPES, pH 7.4). O, open; C, closed.

Effects of Atazanavir on RyR2 pre-treated with low HIV-Tat

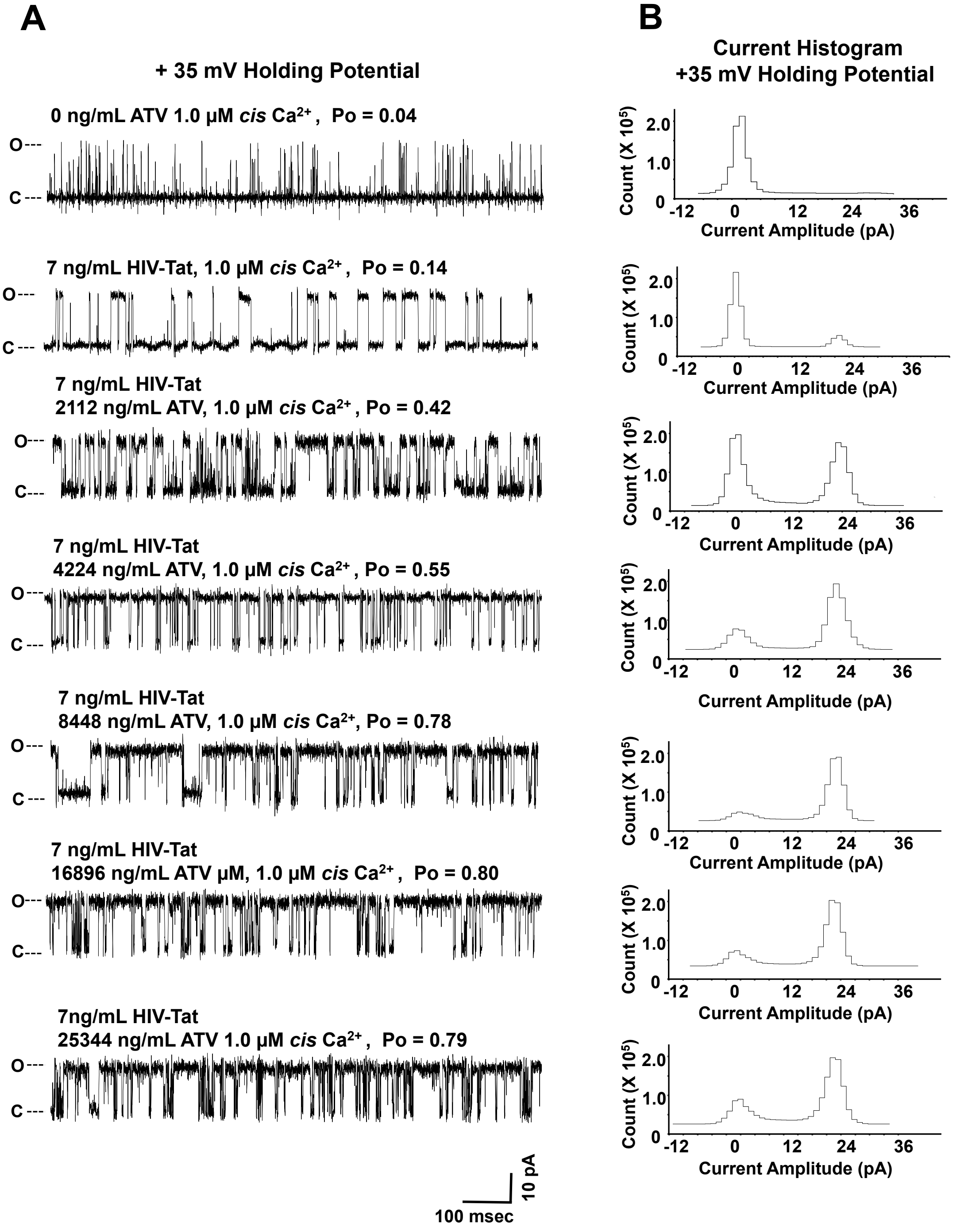

Supplemental Figure S2: Effects of ATV on the open probability of RyR2 that had been pretreated with 7 ng/mL HIV-Tat. Panel A shows a representative 1.0 sec recording of a RyR2 channel with 1.0 µM *cis* Ca^{2+} and before and after pre-treated with 7 ng/mL HIV-tat and varying amount of ATV (representative of n = 10 channels). Single-channel currents were recorded at +35 mV (upward deflections) in symmetric KCl buffer solution (0.25 mM KCl, 20 mM K/HEPES, pH 7.4). O, open; C, closed.

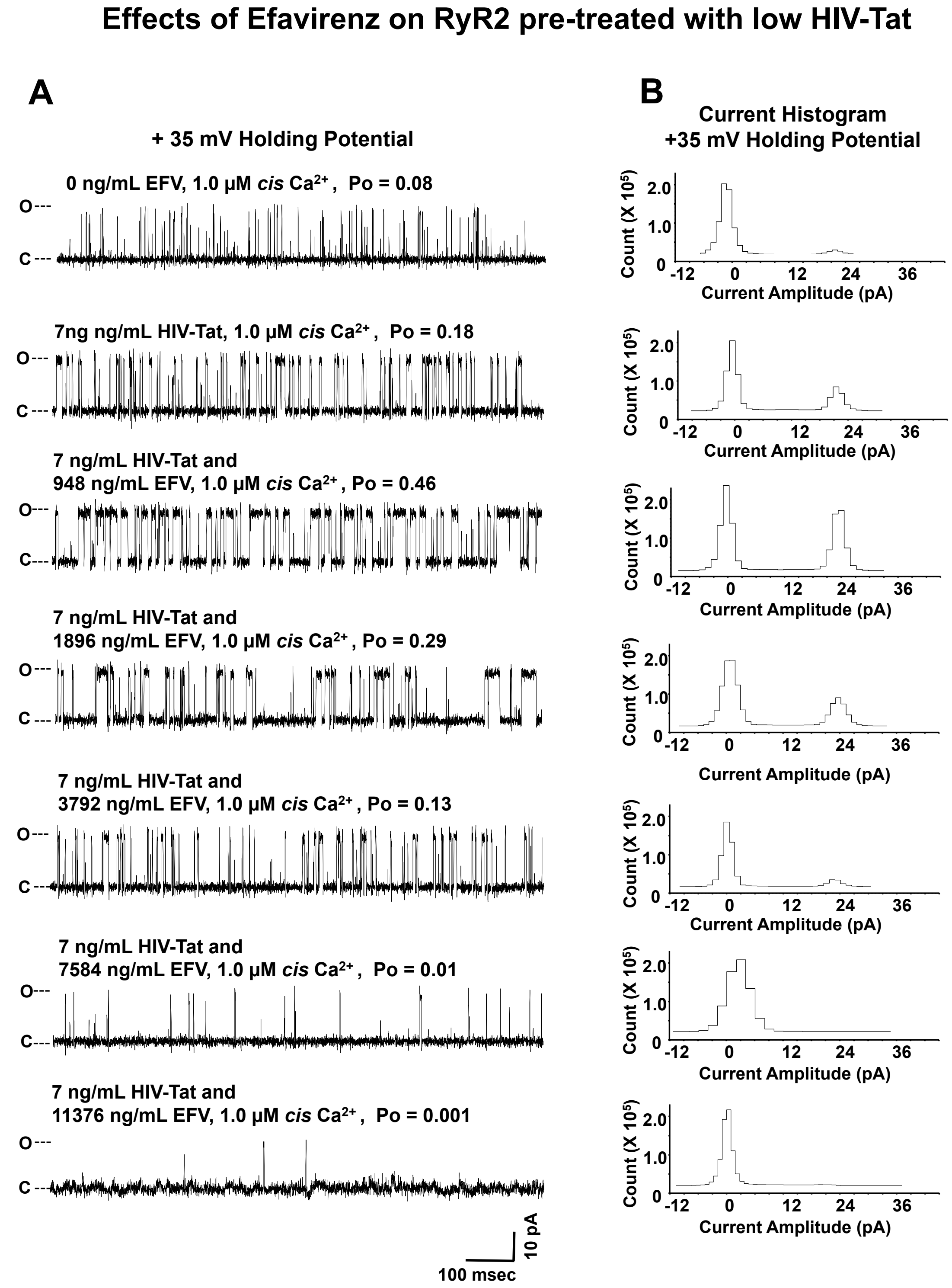

Supplemental Figure S3: Effects of EFV on the open probability of RyR2 that had been pretreated with 7 ng/mL HIV-Tat. Panel A shows a representative 1.0 sec

recording of a RyR2 channel with 1.0 µM *cis* Ca^{2+} and before and after pre-treated with 7 ng/mL HIV-tat and varying amount of EFV (representative of n = 10 channels). Single-channel currents were recorded at +35 mV (upward deflections) in symmetric KCl buffer solution (0.25 mM KCl, 20 mM K/HEPES, pH 7.4). O, open; C, closed.

Effects of ritonavir on RyR2 pre-treated with low HIV-Tat

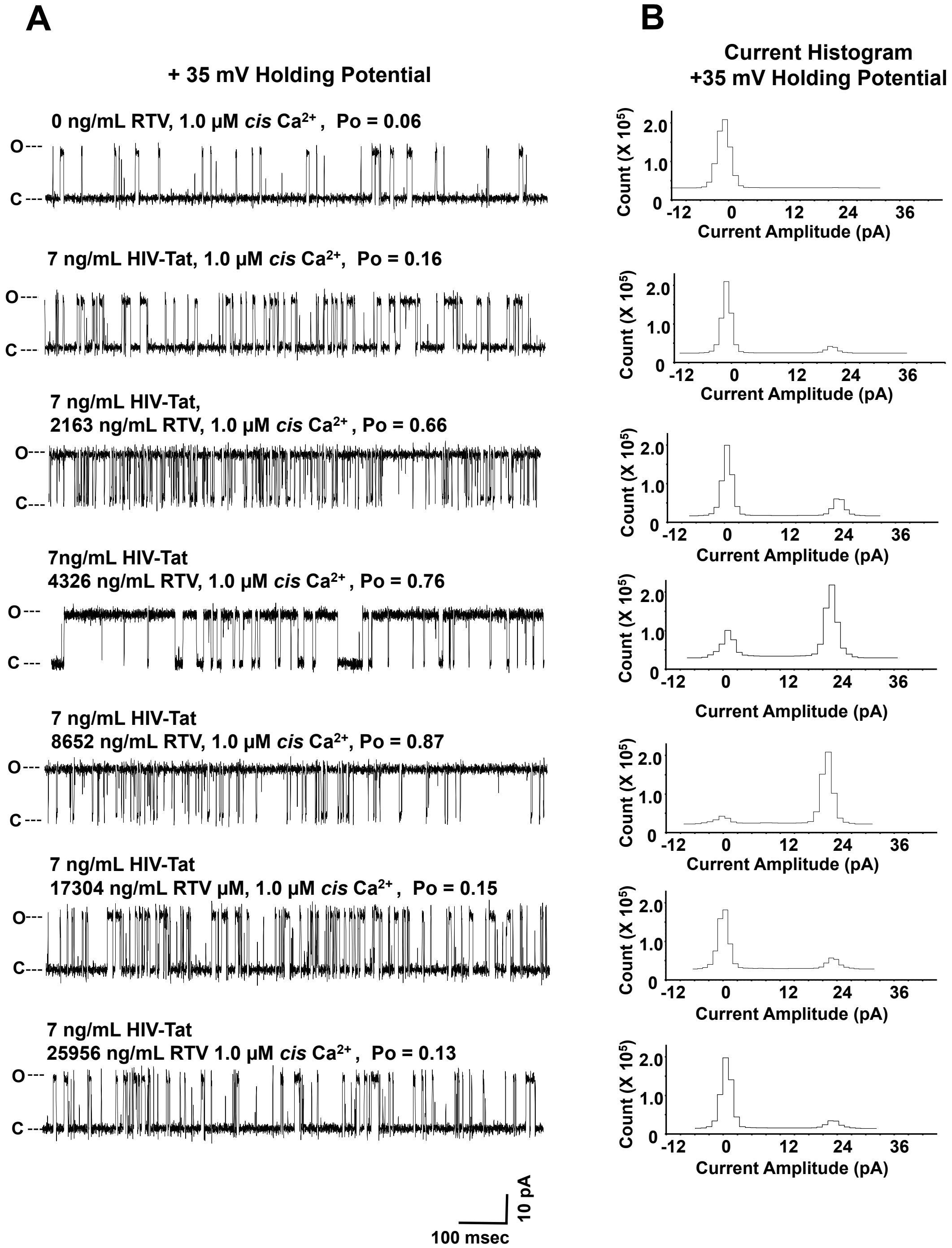

Supplemental Figure S4: Effects of RTV on the open probability of RyR2 that had been pretreated with 7 ng/mL HIV-Tat. Panel A shows a representative 1.0 sec

recordings of a RyR2 channel with 1.0 μM *cis* Ca^{2+} and before and after pre-treated with 7 ng/mL HIV-tat and varying amount of RTV (representative of n = 10 channels). Single-channel currents were recorded at +35 mV (upward deflections) in symmetric KCl buffer solution (0.25 mM KCl, 20 mM K/HEPES, pH 7.4). O, open; C, closed.

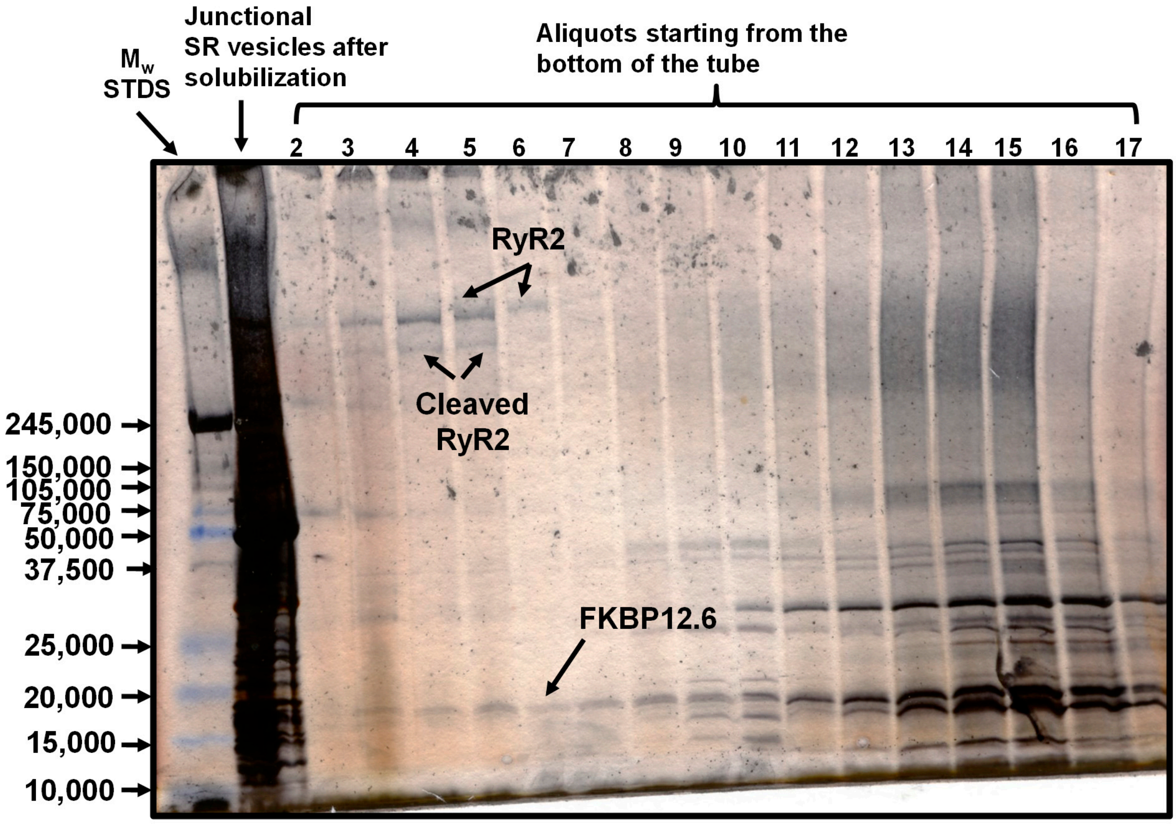

Supplemental Figure S5: Silver stain SDS-PAGE Gel (5-15% linear gradient) showing the protein content of proteoliposomes fractions. Panel A shows a representative silver-stained SDS-PAGE gel profile of solubilized junctional SR vesicles (left) and proteoliposome aliquots after linear sucrose gradient centrifugation, collected from the bottom upwards. Aliquots were mixed with gel dissociation medium and electrophorese on 4-15% linear gradient Tris-glycine polyacrylamide gel for 180 min. Arrows indicate bands corresponding to RyR2, cleaved RyR2 and FKBP12.6. Aliquot #6 was used for all single channel recordings.
